# Structural, Electronic, and Nonlinear Optical Characteristics of Europium-Doped Germanium Anion Nanocluster EuGe*_n_*^−^ (*n* = 7–20): A Theoretical Investigation

**DOI:** 10.3390/molecules30061377

**Published:** 2025-03-19

**Authors:** Chenliang Hao, Xueyan Dong, Chunli Li, Caixia Dong, Zhaofeng Yang, Jucai Yang

**Affiliations:** 1 Inner Mongolia Key Laboratory of Theoretical and Computational Chemistry Simulation, School of Chemical Engineering, Inner Mongolia University of Technology, Hohhot 010051, China; haochenliangyuan@126.com (C.H.); dongxy@imut.edu.cn (X.D.); yangjc@imut.edu.cn (J.Y.); 2 School of Resources and Environmental Engineering, Inner Mongolia University of Technology, Hohhot 010051, China; lichunli16@163.com (C.L.); dongcx@imut.edu.cn (C.D.)

**Keywords:** ground-state structure of Eu-doped germanium clusters, structural evolution patterns, relative stability, simulated photoelectron spectroscopy, nonlinear optical property

## Abstract

Doping rare-earth metals into semiconductor germanium clusters can significantly enhance the stability of these clusters while introducing novel and noteworthy optical properties. Herein, a series of EuGe*_n_*^−^ (*n* = 7–20) clusters and their structural and nonlinear optical properties are investigated via the ABCluster global search technique combined with the double-hybrid density functional theory mPW2PLYP. The structure growth pattern can be divided into two stages: an adsorption structure and a linked structure (when *n* = 7–10 and *n* = 11–20, respectively). In addition to simulating the photoelectron spectra of the clusters, their various properties, including their (hyper)polarizability, magnetism, charge transfer, relative stability, and energy gap, are identified. According to our examination, the EuGe_13_^−^ cluster exhibits a significant nonlinear optical response of the *β*_tot_ value of 7.47 × 10^5^ a.u., and is thus considered a promising candidate for outstanding nonlinear optical semiconductor nanomaterials.

## 1. Introduction

Nanomaterials have emerged as key materials with multidisciplinary potential in various fields, like energy and optics, driven by their unique structure–property relationships [1,2]. In the field of advanced semiconductors, germanium and germanium-based nanomaterials play an essential role [3]. Since 1958—when it was used to develop the world’s first integrated circuit—germanium has gradually been used in various advanced manufacturing fields [4]. For example, germanium has high transparency towards infrared light, allowing it to be used for fabricating infrared lenses and windows [5], narrow-emitting quantum dots for high-color-index displays [6], non-silicon solid-state transistors with high-mobility charge carriers [7], solar cells for high photoelectric conversion efficiency [8], and so on. As next-generation nanomaterials, germanium and germanium-based semiconductors are gradually becoming irreplaceable in cutting-edge technologies and high-precision manufacturing. Additionally, more and more unique physical and chemical properties await further discovery, so it is urgent to develop novel germanium-based semiconductor materials to further satisfy current industrial and societal needs.

Pure germanium nanoclusters provide excellent semiconductor performance and are already widely applied in mainstream advanced optoelectronic devices. However, pure germanium has a large number of dangling bonds, leading to a highly sensitive device service environment [9,10]. To overcome this situation and prolong the devices’ lifetimes, one effective and easy-to-implement strategy is doping transitional metal (TM) into pure germanium nanoclusters. There are several examples of this in the literature. Manganese-doped anionic germanium nanoclusters MnGe_6_^−^ and Mn_2_Ge_7_^−^ were studied by means of joint photoelectron spectroscopy (PES) with theoretical calculations, and the results showed that MnGe_6_^−^ and Mn_2_Ge_7_^−^ have the same symmetry of *C*_2*v*_ and vertical electron detachment energies of 2.58 ± 0.08 and 2.88 ± 0.08 eV, respectively [11]. A series of transitional and lanthanide metals, including Sc, Ti, V, Y, Zr, Nb, Lu, Hf, and Ta, were examined by means of anion PES at a 213 nm pulse using a Nd^3+^:YAG laser, which revealed a Ge_16_ cage structure with a large cavity, allowing the metal to be encapsulated [12]. In a theoretical investigation, bimetallic Mo_2_Ge*_n_* (*n* = 9–15) clusters were studied via the B3LYP density functional approach, and the Mo_2_Ge_9_ theoretical predicted structure was analogous to the one that was experimentally observed [13]. Group 4 and 5 elements in germanium clusters TMGe_8–17_^−^ were studied by applying a genetic algorithm coupled with density functional theory. The calculated PES highly agreed with the measured spectra, in which the complete closed cage motif showed great stability [14]. Monoanionic Ru_2_Ge*_n_*^−^ (*n* = 3–20) was examined by using a genetic algorithm associated with PBE functionality. The ground-state structure study showed that, when extra electrons were added in, the cluster structure was significantly influenced [15]. Regarding noble metal-doped germanium nanoclusters, AuGe_12_ clusters with a bicapped pentagonal prism geometry were investigated via density functional theory calculations. The results showed that the hybridization between the transitional metal Au and the germanium cage enhanced the chemical stability of the whole cluster [16]. Such conclusions were also made for IrGe*_n_*^−^ (*n* = 1–20) clusters [17]. Because of the similar chemical and physical properties of silicon and germanium, Kumar and Kawazoe, using the ab initio pseudopotential plane wave method, calculated the structures of transitional metal Ti-, Zr-, and Hf-doped Ge_16_, forming the Frank–Kasper structure MGe_16_ and capped decahedral or cubic MGe_16_ and MGe_14_; moreover, they found that the growth behaviors were different from those for the MSi*_n_* nanocluster [18]. Another research study by Han and Hagelberg proved that TMSi*_n_* with *n* = 12 or *n* = 16 and TMGe*_n_* with *n* = 10, *n* = 12, or *n* = 16 are the most promising candidates for modular nanomaterials [19]. Besides doping with *d*-block transition metals, another way to achieve such goals is through doping with rare-earth metals. A scandium atom doped into an anion germanium cluster can vastly enhance the stability of the cluster, owing to the form of high-symmetry Frank–Kasper ScGe_16_^−^ with 68 valence electron-filled shell configurations [20,21,22,23]. The different cage types of ScGe_12_ were also theoretically studied [24]. Li et al. systematically explored the structural evolution, relative stability, and electronic properties of LaGe*_n_*^−^ (*n* = 3–14), and their results indicated that a lanthanum atom doped into a pure germanium cluster increased the interactions between germanium atoms and enhanced the stability of the whole system; such building blocks have potential for the design of molecular chains [25]. A series of germanium anionic clusters ReGe_6_^−^ doped with the heavy rare-earth elements Gd, Tb, Dy, Ho, Er, Tm, Yb, and Lu were identified using the PBE0 functional method combined with the def2-TZVP basis set. The calculations showed that rare-earth atoms doped into center pentagonal ring structures have greater stability and aromaticity than other types, making them potential building blocks for novel rare-earth-based semiconductor materials [26]. The lowest energy structures of rare-earth element Yb-, Pm-, and Dy-doped neutral and cationic germanium clusters Ge*_n_*M (*n* = 9, 10; M = Yb, Pm, and Dy) were determined using a genetic algorithm coupled with the PBE functional and projected augmented wave method. The results showed that rare-earth metal doping into germanium is favorable in terms of the energy direction [27]. With the great number of potential applications in the current semiconductor industry and the huge demand in today’s society, there is an urgent need to discover novel, stable, and ultra-efficient nanomaterials.

The element europium (Eu) is a typical and unique luminescent activator, widely used in a variety of luminescent materials. Depending on the coordination environment and electron transition of its 4*f*-4*f* and 4*f*-5*d* orbitals, it can exhibit different optical properties, and there is high demand for its application in different situations. In view of this, we selected Eu as a dopant in pure germanium clusters to design novel semiconductor building blocks. The ground-state structures of the EuGe*_n_*^−^ (*n* = 7–20) clusters were systematically determined through stepwise optimization, and their nonlinear optical properties were investigated. Moreover, their photoelectron spectra, magnetic behavior, relative stability, energy gap, and density of states were all studied. The results reveal the large polarizability and hyperpolarizability of the EuGe_13_^−^ clusters, which may serve as potential nonlinear optical materials for the next-generation semiconductor industry.

## 2. Results and Discussion

### 2.1. Ground-State Structure and Evolution Pattern of EuGe_n_^−^ (n = 7–20) Nanoclusters

**Figure 1:**
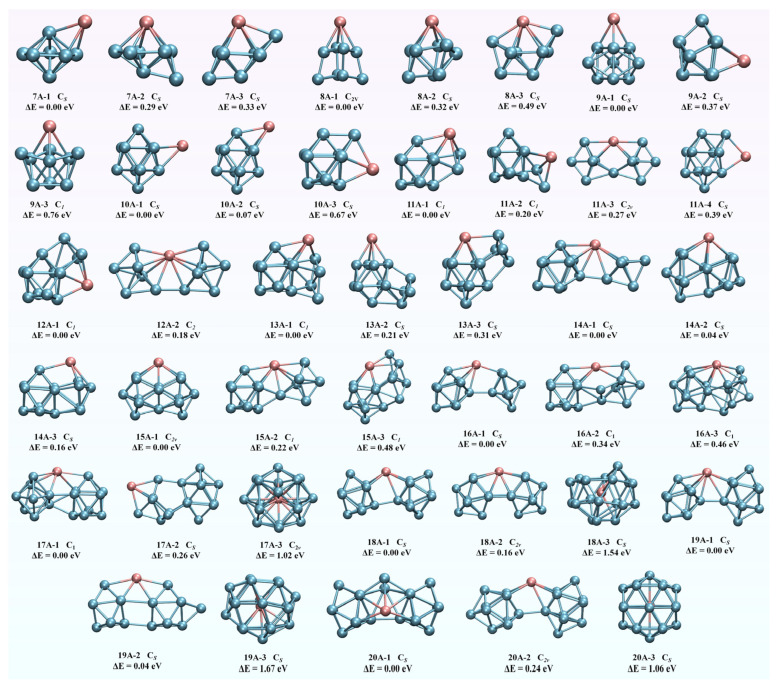
The lowest-energy structures and other isomers of EuGe*_n_*^−^ (*n* = 7–20) after mPW2PLYP method optimization. Pink and cyan represent Eu and Ge atoms, respectively.

The ground-state structures, symmetry, and energy differences in EuGe*_n_*^−^ (*n* = 7–20) optimized at the mPW2PLYP level are shown in Figure 1, and the Cartesian coordinates of each cluster are collected in Table S1. To further ensure the ground-state structures, the AIMD results are shown in Figure S1 (Supporting Information), and the total energies at mPW2PLYP with zero-point energy at TPSSh are collected in Table S2. Each cluster is noted as *n*A-*x*, where *n* is the number of Ge atoms, A is the anionic cluster, and x is the sequence number from small to large for increasing energy differences compared to the energy of the ground-state structure. The spin multiplicities of EuGe*_n_*^−^ (*n* = 7–20) clusters were all predicted for nonets. For EuGe_7_^−^, **7A-1** and **7A-2** have the same symmetry of *C_s_*, which can be seen as one Eu atom replacing one Ge atom in the pure Ge_8_ cluster [28]. The structure of **7A-3** is two Ge atoms forming a capped tetragonal bi-pyramid, of which the Eu atom is the vertex. The energies of **7A-2** and **7A-3** are higher than that of **7A-1**, by 0.29 and 0.33 eV, respectively. For EuGe_8_^−^, **8A-1** is one Eu atom adsorbed on the top of the square frustum of Ge_8,_ and **8A-2** is a typical tricapped triangular prism (TTP) structure where the Eu atom is located on one side of the triangular prism. The structure of **8A-3** can be described as one extra Ge atom adsorbed onto **7A-3**. The energy differences for **8A-2** and **8A-3** with regard to **8A-1** are 0.32 and 0.49 eV, respectively. For EuGe_9_^−^, all isomers have a similar structure, depicted as one Eu atom and two Ge atoms capped on the TTP Ge_6_ basic framework, where the difference is only in the three atoms’ cap location. **9A-1** is more stable than **9A-2** and **9A-3**, with energy differences of 0.37 and 0.76 eV, respectively. For EuGe_10_^−^, **10A-1** and **10A-2** are similar types, with one Eu atom site on the side of two capped anti-prisms Ge_10_. Due to the similarity of their structures, the energies of **10A-1** and **10A-2** are close, with a difference of only 0.07 eV. **10A-3** is derived by adding one Ge atom to the ground-state structure of EuGe_9_^−^. The energy of **10A-3** is higher than that of **10A-1** by about 0.67 eV. For EuGe_11_^−^, a linked structure emerges. The isomers of **11A-1** and **11A-2** are all depicted with Eu atoms as linkers between two parts of TTP Ge_9_ and Ge_2_. The structure of **11A-3** is one Eu atom and one Ge atom on the plane of *C_s_* symmetry, co-connected to two trigonal bi-pyramids Ge_5_. **11A-4** can be seen as one extra Ge atom added onto the side of **10A-1**. The energy differences for **11A-2**, **11A-3**, and **11A-4** with regard to **11A-1** are 0.20, 0.27, and 0.39 eV, respectively. For EuGe_12_^−^, the lowest-energy **12A-1** is a linked type with a Eu atom connected to Ge_3_ as one part and one capped anti-prism Ge_9_ as the other part. The structure of **12A-2** exhibits a center Eu atom joining two tetragonal bi-pyramids Ge_6_. **12A-1** is more stable than **12A-2**, with an energy difference of 0.18 eV. For EuGe_13_^−^, the minimum-energy structure is a Eu atom associated with TTP Ge_9_ and a four-membered ring Ge_4_. **13A-2** and **13A-3** are similar motifs viewed as a four-membered ring Ge_4_ sub-cluster adsorbed onto two capped anti-prism basic frameworks; their energies are higher than that of the **13A-1** linked structure by 0.21 and 0.31 eV, respectively. For EuGe_14_^−^, all selected isomers are of linked forms. **14A-1**, with *C_s_* symmetry, consists of a Eu atom connected to one unit of TTP Ge_9_ and one unit of trigonal bi-pyramid Ge_5_. **14A-2** is an energy-degenerate form of **14A-1** showing one sub-structure of TTP Ge_9_ and another of square-pyramid Ge_5_. The structure of **14A-3** shows mirror symmetry with that of **14A-2**, but with a slight difference. The energy difference between **14A-1** and **14A-2** is only 0.04 eV, and that between **14A-1** and **14A-3** is 0.16 eV. For EuGe_15_^−^, the lowest-energy structure of **15A-1** with *C*_2*V*_ symmetry can be divided into one Eu atom combined with three Ge atoms forming a co-planar quaternary ring that connects two mirror-symmetric pentagonal pyramids Ge_6_. **15A-2** and **15A-3** can both be described as a Eu atom binding one sub-unit of TTP Ge_9_ and one sub-unit of Ge_6_. The energies of **15A-2** and **15A-3** are higher than that of **15A-1** by 0.22 and 0.48 eV. For EuGe_16_^−^, the architectures of **16A-1**, **16A-2**, and **16A-3** can all be noted as a Eu atom as a connector joining one sub-cluster of TTP Ge_9_ and another sub-cluster of Ge_7_. The energy of **16A-1** is lower than those of **16A-2** and **16A-3** by 0.34 and 0.46 eV, respectively. For EuGe_17_^−^, the skeleton of **17A-1** can be described as a center Eu atom bridging one capped anti-prism Ge_9_ and one anti-prism Ge_8_. **17A-2** is of a chain type with two parts of two Ge atoms capping an anti-prism Ge_10_ and two Ge atoms capping a tetragonal bi-pyramid where the Eu atom is not located at the middle position as a linker. The energy of **17A-2** is 0.26 eV higher than that of **17A-1**. The cage structure of **17A-3** has a larger energy difference relative to the linked type of **17A-1** of about 1.02 eV. For EuGe_18_^−^, **18A-1** and **18A-2** are analogous structures portrayed as a Eu atom anchoring two TTP Ge_9_ sub-clusters. **18A-3** is an endohedral cage structure where a Eu atom is encapsulated in a Ge_18_ cage skeleton. The energies of **18A-2** and **18A-3** are 0.16 and 1.54 eV higher than the energy of **18A-1**. For EuGe_19_^−^, **19A-1** and **19A-2**, with the same *C_s_* symmetry, can be viewed as a Eu atom bridging one Ge atom capping a Ge_9_ anti-prism on one side and two Ge atoms capping a Ge_10_ anti-prism on the other. The energies of **19A-1** and **19A-2** are degenerative with a small energy difference of 0.04 eV. The **19A-3** cage structure originates from the cage motif of **17A-3** with the addition of two extra Ge atoms into the Ge bones. The energy of **19A-3** is 1.67 eV larger than that of **19A-1**. For EuGe_20_^−^, the energy-minimum pattern **20A-1** can be deconstructed as a middle triangle composed of one Eu and two Ge atoms that connects symmetrical bi-lateral TTP Ge_9_ blocks. The geometry of **20A-2** is a Eu atom located at the center, bridging two capped anti-prisms. The energies of **20A-2** and the cage structure **20A-3** are 0.24 and 1.06 eV larger than that of **20A-1**.

To sum up, the structural evolution of EuGe*_n_*^−^ (*n* = 7–20) can be classified into two stages: (i) for small-sized clusters with *n* = 7–10, the configurations mainly present an adsorbed mode, where a Eu atom is adsorbed onto the Ge*_n_*^−^ cluster; (ii) for medium-sized clusters with *n* = 11–20, the structures exhibit a pronounced tendency to form elongated link structures. Specifically, Eu atoms serve as connection points, bridging sub-clusters with TTP structures of Ge_9_ and Ge*_n_*_-9_. Consequently, the EuGe_9_ sub-cluster, which is formed via linkage between a Eu atom and a TTP-structured Ge_9_ unit, constitutes the fundamental structural unit for the growth of medium-sized clusters.

### 2.2. Predicted Photoelectron Spectroscopy of EuGe_n_^−^ (n = 7–20)

**Figure 2:**
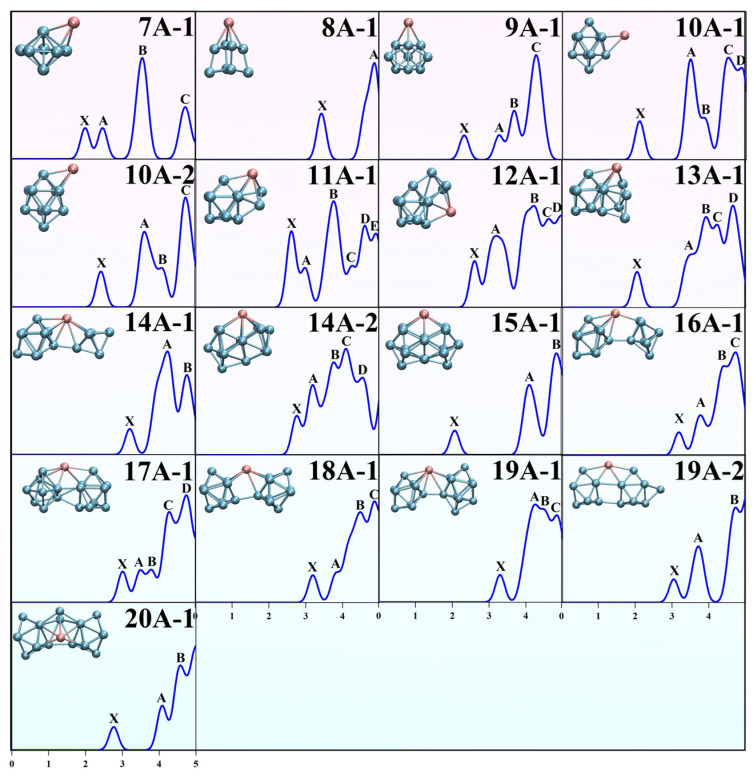
Simulated PES of EuGe*_n_*^−^ (*n* = 7–20).

Photoelectron spectroscopy (PES) is a common technique for indirectly testing the geometry and electronic structure of nanoclusters. Because nanoclusters have multiple isomers and states that are difficult to accurately determine, PES characterization of nanoclusters is absolutely crucial. In view of this, the theoretical predicted PES of EuGe*_n_*^−^ (*n* = 7–20) was calculated via the method of Koopmans’ theorem and the first peak equivalent to vertical detachment energy (VDE) calculated under the mPW2PLYP level. The first ionization potential (IP) is defined as E(anion) − E(neutral). The first peak is set to the first IP + E(HOMO); then, the second peak is obtained by adding the absolute value of the energy difference between the occupied orbital below the HOMO and the HOMO orbital as the second IP, and so on. The number of peaks and their position on the PES reveal the energy differences between diverse *N*-1 states and the original *N*-electron state. By default, all orbitals have a strength equal to 1.0, where peak broadening can be plotted by means of strength stacking and Gaussian broadening. A plot for each cluster is shown in Figure 2, with a Gaussian broadening of 0.30 eV for the energy region smaller than 5 eV. The peaks of each EuGe*_n_*^−^ (*n* = 7–20) cluster with energy from low to high are marked as X and A to E in alphabetical order. For EuGe_7_^−^, the PES results exhibit four peaks at 2.00 (X), 2.47 (A), 3.55 (B), and 4.71 (C) eV. For EuGe_8_^−^, in the energy region smaller than 5 eV, there are only two peaks located at 3.43 (X) and 4.87 (A) eV. For EuGe_9_^−^, there are four peaks (X, A to C) located at 2.33, 3.28, 3.69, and 4.29 eV (respectively). For EuGe_10_^−^, the PES results for the ground-state structure **10A-1** and its energy-degenerate isomer **10A-2** are shown. They have similar shapes and positions of their X, A, and B peaks due to the similarity of their structures. The PES results for **10A-1** show five peaks in the energy region from 0 to 5 eV, sited at 2.10, 3.49, 3.85, 4.51, and 4.87 eV, compared with those for **10A-2** showing only four peaks at 2.42, 3.60, 4.06, and 4.71 eV; thus, they can be distinguished by the number of peaks. For EuGe_11_^−^, the six peaks X and A to E are located at 2.63, 2.99, 3.77, 4.28, 4.62, and 4.92 eV, respectively. For EuGe_12_^−^, three obvious peaks, X, A, and B, are located at 2.61, 3.21, and 4.23 eV, with peaks C and D as tail peaks of B at 4.64 and 4.98 eV. For EuGe_13_^−^, three clear spikes, X, B, and D, are located at 2.05, 3.92, and 4.66 eV. A and C are shoulder peaks of B at 3.50 and 4.22 eV. For EuGe_14_^−^, the PES curves of the two near-energy structures **14A-1** and **14A-2** have different numbers of peaks and shapes, making them easy to distinguish. The peaks X, A, and B of **14A-1** are positioned at 3.21, 4.23, and 4.76 eV. The PES results for **14A-2** show two more peaks than those for **14A-1**, at 2.76 eV for A, 3.20 eV for B, 3.77 eV for C, 4.10 eV for D, and 4.55 eV for E. For EuGe_15_^−^, there are three independent peaks at 2.07 eV (A), 4.09 eV (B), and 4.83 eV (C). For EuGe_16_^−^, four consecutive peaks, X and A to C, appear at positions of 3.20, 3.78, 4.43, and 4.74 eV. For EuGe_17_^−^, five peaks, X and A to D, are located at 3.02, 3.51, 3.79, 4.28, and 4.75. Among them, the peaks A and B are very close to each other. For EuGe_18_^−^, three evident peaks, X, B, and C, are sited at 3.19, 4.48, and 4.87 eV. A is a satellite peak in the middle of X and B at 3.84 eV. For EuGe_19_^−^, the PES results for **19A-1** present two clear peaks, X at 3.30 eV and A at 4.26, with tailed peaks at the end of the energy region denoted as C at 4.52 and D at 4.84 eV. The PES results for **19A-2**, compared with those for **19A-1**, only show three peaks at energy positions of 3.05, 3.72, and 4.73 eV, so they can be discerned by the curve shape and number of peaks. For EuGe_20_^−^, in the selected energy region, there are three peaks at 2.77, 4.09, and 4.58 eV. Because there are no comparable experimental results, to some extent, these predicted PES results can provide a reference and instructions for future experimental investigations.

### 2.3. Magnetic Moments and Charge Transfer of EuGe_n_^−^ (n = 7–20)

The Eu atom possesses remarkable magnetism as a result of the spin magnetic moment of electrons in its 4*f* orbitals. When a Eu atom is doped into a pure germanium cluster, it brings this novel magnetic property to the nanocluster. Considering this, a natural population analysis (NPA) in the NBO 3.1 software was performed to discover the charge, valence electron configuration, and magnetic moments of each orbital and the total structure of EuGe*_n_*^−^ (*n* = 7–20). The data are collected in Table 1. From Table 1, it can be seen that the Eu atoms in all ground-state structure clusters have a positive charge of from 0.51 a.u. to 0.81 a.u., which means that the Eu atoms in the clusters serve as electron donors and the Ge frameworks serve as electron acceptors. Between the Eu and Ge atoms, ionic bonds are formed. When *n* = 7–10, the ground-state structure is replaced by a structure in which the Eu atom has a valence electron configuration of 6*s*^0.80–1.00^4*f*^6.98^5*d*^0.18–0.41^6*p*^0.14–0.23^. When the ground-state structure changes to a linked type (*n* = 11–20), the valence electron configuration of Eu becomes 6*s*^0.24–0.51^4*f*^6.97^5*d*^0.63–0.85^6*p*^0.15–0.32^. From the valence electron configuration of the Eu atom of Xe [6*s*^2^4*f*^7^], it is known that the 4*f* electrons of the Eu atom in EuGe*_n_*^−^ (*n* = 7–20) do not participate in bonds, mainly providing magnetic moments for the cluster. Without bonding involving the 4*f* electrons of Eu, the bonding between Eu and Ge is mainly attributed to electrons in Eu’s 5*d* and 6*s* orbitals hybridizing with electrons in Ge’s 4*s* and 4*p* orbitals.

**Figure 3:**
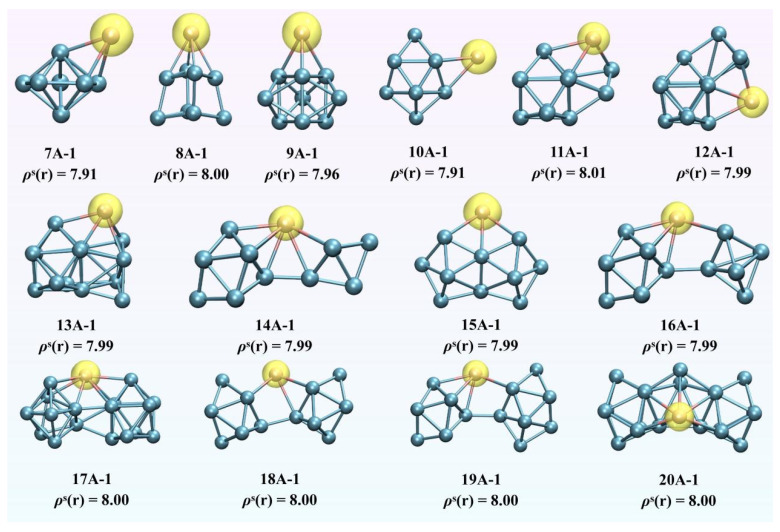
Spin density contour maps of EuGe*_n_*^−^ (*n* = 7–20) clusters.

To further explore the magnetism of EuGe*_n_*^−^ (*n* = 7–20), the spin density and spin population were examined. The spin density three-dimensional contour maps of EuGe*_n_*^−^ (*n* = 7–20) shown in Figure 3 were obtained using the following equation:

(1) $$\rho^{s}(r)=\rho^{\alpha }(r)-\rho^{\beta }(r)$$

The equation definition is the electron density of Alpha minus the electron density of Beta in three-dimensional space. It can be easily seen that the iso-surfaces of spin density are all located in the Eu atom, and the values of $\rho^{s}(r)$ are all close to or equal to 8, indicating that the magnetism of EuGe*_n_*^−^ (*n* = 7–20) originates from the Eu atom. A further analysis was carried out via the spin population method, as shown in Table 1. For *n* = 7–10, the adsorption structure stage, the total magnetic moment contribution by the Eu atom is 7.86 to 7.95 μ*_B_*, with a contribution ratio exceeding 99.10%. For *n* = 11–20, the linked stage, the total magnetic moment provided by the Eu atom is 7.11 to 7.48 μ*_B_*, with a contribution ratio exceeding 88.96%—a slight decrease compared to that of the adsorption structure.

### 2.4. Relative Stability of EuGe_n_^−^ (n = 7–20)

The average binding energy (*ABE*) is an important parameter used to describe the relative stability with the change in the size of a cluster. The second-order energy difference (Δ^2^*E*) is another critical parameter used to evaluate the stability within one cluster of its left and right sides. The *ABE* and Δ^2^*E* are calculated through following Equations (2) and (3):

(2) $$ABE\left({\mathrm{EuGe}}_{n}^{-}\right)=\frac{\left[\left(n-1\right)E\left(\mathrm{Ge}\right)+E({\mathrm{Ge}}^{-})+E(\mathrm{Eu})-E\left({\mathrm{EuGe}}_{n}^{-}\right)\right]}{n+1}$$

(3) $$\Delta^{2}E{(\mathrm{EuGe}}_{n}^{-})=E({\mathrm{EuGe}}_{n-1}^{-})+E({\mathrm{EuGe}}_{n+1}^{-})-2E({\mathrm{EuGe}}_{n}^{-})$$

**Figure 4:**
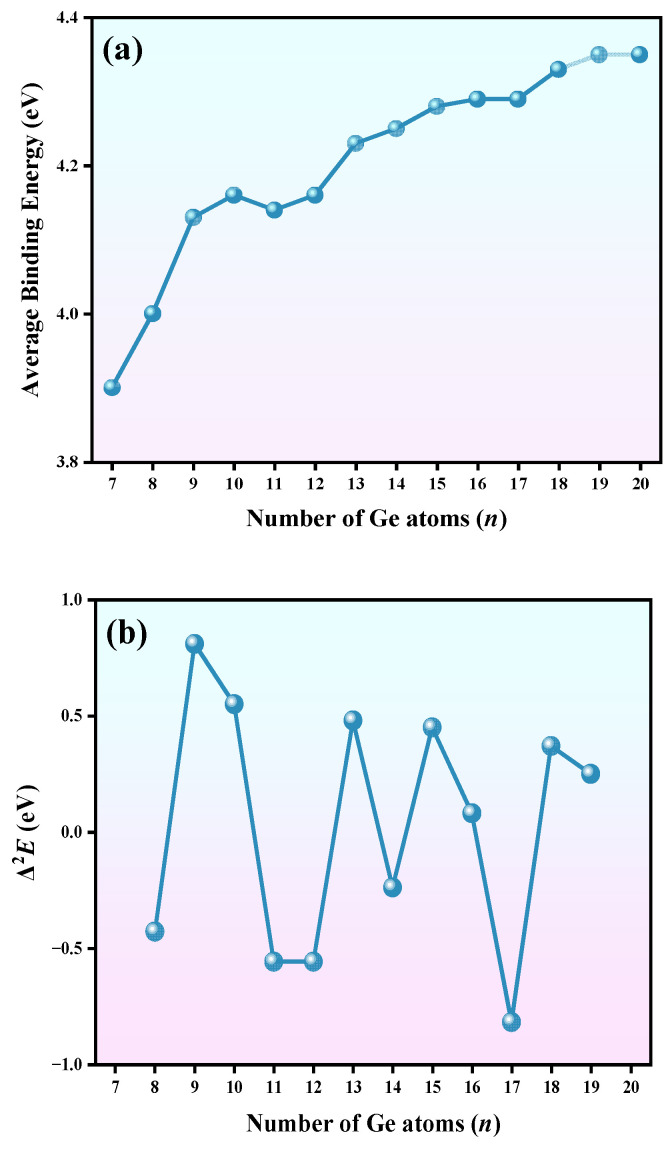

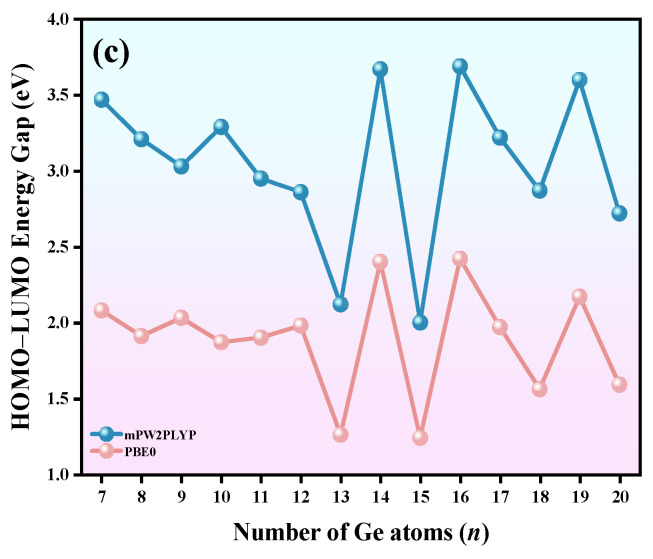
The (**a**) average binding energy (*ABE*, in eV), (**b**) second-order energy difference (Δ^2^*E*, in eV), and (**c**) HOMO–LUMO gap (in eV) of EuGe*_n_*^−^ (*n* = 7–20) clusters.

The values of *ABE* and Δ^2^*E* are plotted in Figure 4. From Figure 4a, in the adsorption pattern phase, the *ABE* rises sharply, with a peak observed for EuGe_10_^−^ of 4.16 eV. At the linked structure stage, two obvious sections can be seen. When *n* = 11–17, the increasing trend is smaller than that for the adsorption stage, tending toward a flat curve for *n* = 18–20. The maximum and minimum values of the *ABE* within the cluster size were found for EuGe_19_^−^ at 4.35 eV and EuGe_7_^−^ at 3.90 eV. In Figure 4b, the Δ^2^*E* curve presents four peaks at 0.81, 0.48, 0.45, and 0.37 eV, belonging to EuGe_9_^−^, EuGe_13_^−^, EuGe_15_^−^, and EuGe_18_^−^; this means that these clusters are more stable than their adjacent ones.

The energy gap (*E*_g_) is a very important physical quantity that denotes the energy difference between the lowest unoccupied molecular orbital and the highest occupied molecular orbital, described in Equation (4):

(4) $$E_{g}=E(\mathrm{LUMO})-E(\mathrm{HOMO})$$

The *E*_g_ values in Figure 4c were calculated using the mPW2PLYP and PBE0 methods. In general, the number of Hartree–Fock components in a functional strongly affects the value of *E*_g_. The mPW2PLYP functional has more Hartree–Fock components and usually over-estimates *E*_g_. However, having fewer Hartree–Fock components in a functional mostly leads to under-estimation of *E*_g_, such as with the pure PBE functional. In view of this, the hybrid density functional PBE0 was implemented to calculate *E*_g_. The trends in *E*_g_ values calculated using mPW2PLYP and PBE0 are generally similar, being only slightly different at *n* ≤ 11; when *n* > 11, the two *E*_g_ curves present the same trend. The maximum *E*_g_ values pertain to EuGe_14_^−^, with 3.67 eV at the mPW2PLYP level and 2.40 eV at the PBE0 level. The minimum *E*_g_ values belong to EuGe_15_^−^, with 2.00 eV at the mPW2PLYP level and 1.24 eV at the PBE0 level. The above results show that the EuGe*_n_*^−^ (*n* = 7–20) clusters are all semiconductors and potential semiconductor building block candidates for next-generation optoelectronic materials.

### 2.5. Nonlinear Optical Properties of EuGe_n_^−^ (n = 7–20)

The Eu atom is a typical optical activator. In previous works [29,30], lone-pair electrons helped to enhance the nonlinear optical effects of optical materials. The Eu atom has seven single electrons in its 4*f* orbitals; when an extra electron is brought into the cluster, the whole cluster changes to have eight single electrons. In view of this, the dipole moment (*μ*_0_), static polarizability (*α*_0_), static first hyperpolarizability (*β*_tot_), components of *β*_tot_ in the x, y, and z directions, and projection of *β*_tot_ in the direction of the dipole moment (*β*_prj_) for EuGe*_n_*^−^ (*n* = 7–20) clusters were calculated at the level of long-range corrected density functional theory CAM-B3LYP, which is commonly used to evaluate nonlinear optical responses [31,32]. The results are summarized in Table 2. For clusters with *n* = 7–10, the dipole moment varies between 0.91 and 2.02 a.u. When *n* increases to the range of 11 to 20, the dipole moment exhibits a significant increase, ranging from 2.27 to 3.94 a.u., which can be attributed to changes in the cluster configuration. The static polarizability of the ground-state structures of EuGe*_n_*^−^ (*n* = 7–20) demonstrates an overall increasing trend, with particularly notable values at *n* = 13 and 20, reaching 803.99 and 860.61 a.u., respectively. The second-order nonlinear optical coefficient of the clusters, i.e., the first hyperpolarizability, can reflect the nonlinear optical response of the materials. Thus, the static hyperpolarizability and its components along the three directions of the 14 ground-state clusters were simulated and compared. It was observed that the *β*_tot_ value for EuGe_13_^−^ was markedly higher than those of the other clusters, reaching 747,032.61 a.u., with *β*_yyy_ being the predominant contributing component. These findings suggest that EuGe_13_^−^ holds significant potential for applications in the development of nonlinear optical materials.

**Figure 5:**
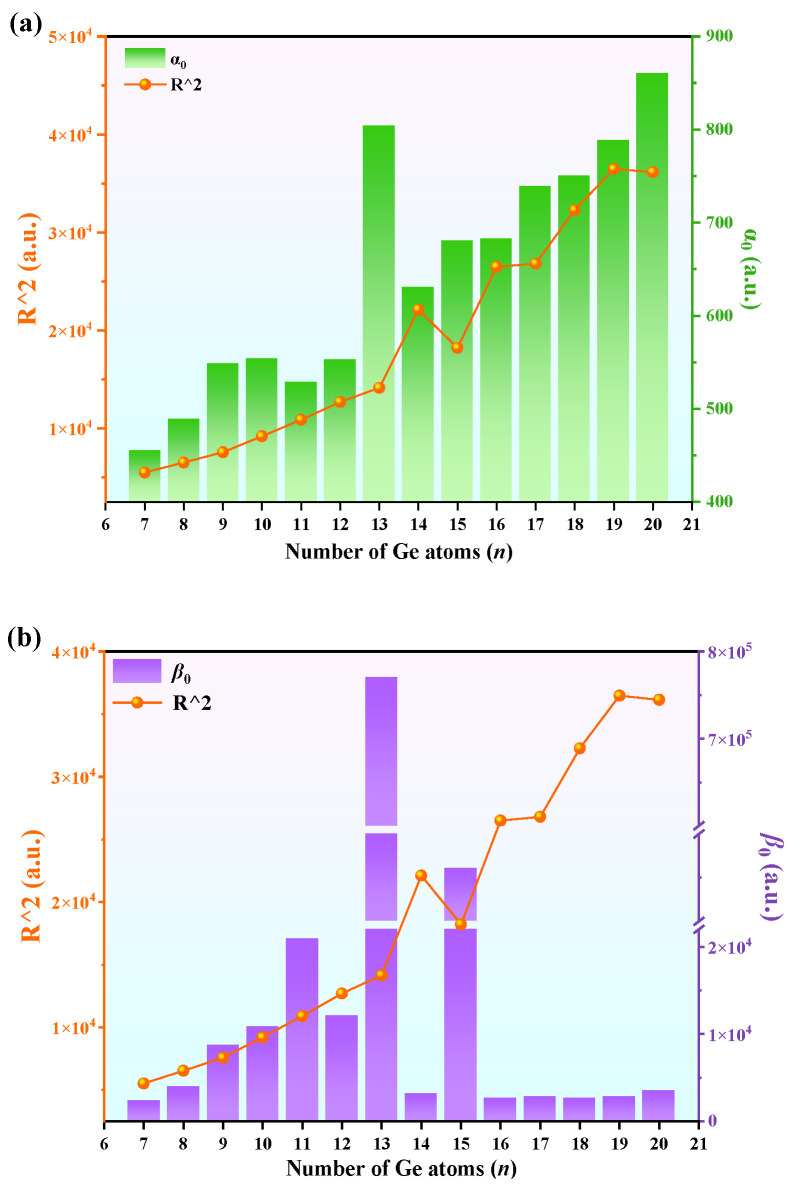
The relative relationships between (**a**) *α*_0_ and R^2^ and (**b**) *β*_tot_ and R^2^.

To further elucidate the nonlinear optical properties of EuGe*_n_*^−^ (*n* = 7–20), the electronic spatial extent R^2^ of the clusters serves as an effective descriptor quantifying their polarizability and hyperpolarizability [33]. Figure 5a,b, respectively, illustrate the relative relationship between *α*_0_ and R^2^ and that between *β*_tot_ and R^2^ as the number of Ge atoms increases. From Figure 5a, it can be seen that the *α*_0_ value increases as the cluster size and R^2^ increase. However, for *β*_tot_, there is a linear trend only for small-sized clusters with *n* < 12 (shown in Figure 5b). Notably, the polarizability reaches a significant peak when *n* = 13.

**Figure 6:**
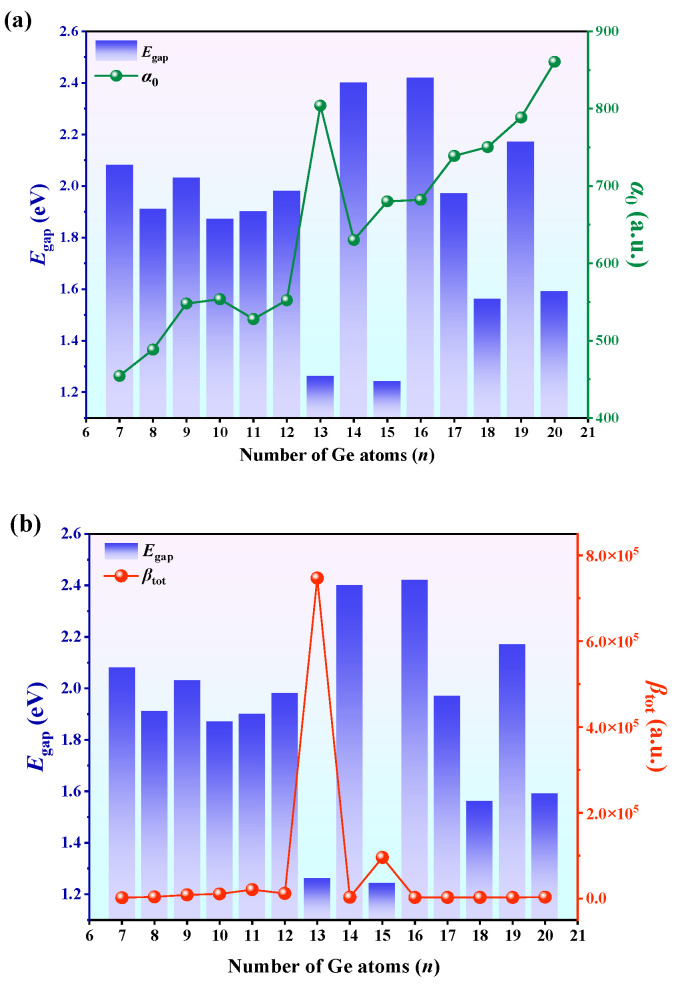
The relationships of (**a**) static polarizability (*α*_0_) and (**b**) static hyperpolarizability (*β*_tot_) with the HOMO–LUMO energy gap (*E*_g_) of EuGe*_n_*^−^ (*n* = 7–20) clusters.

Besides the descriptor R^2^, the relative relationships between the polarizability, hyperpolarizability, and energy gap are illustrated in Figure 6a,b. In the figure, the HOMO–LUMO gap exhibits a negative correlation with *α*_0_ and *β*_tot_. Smaller *E*_g_ values could account for the larger *α*_0_ and *β*_tot_ observed for EuGe_13_^−^ and EuGe_15_^−^. Given that the symmetry of EuGe_15_^−^clusters is higher than that of EuGe_13_^−^ clusters, the electron cloud distribution in the EuGe_15_^−^ clusters is more uniform, rendering them less susceptible to polarization by external electric fields. Consequently, the α_0_ and *β*_tot_ values for EuGe_15_^−^ clusters are lower than those for EuGe_13_^−^ clusters. Additionally, the calculated *β*_prj_ values can serve as a reference and comparison for hyperpolarizability values obtained from future experiments on electric field-induced second harmonic generation (EFISH).

**Figure 7:**
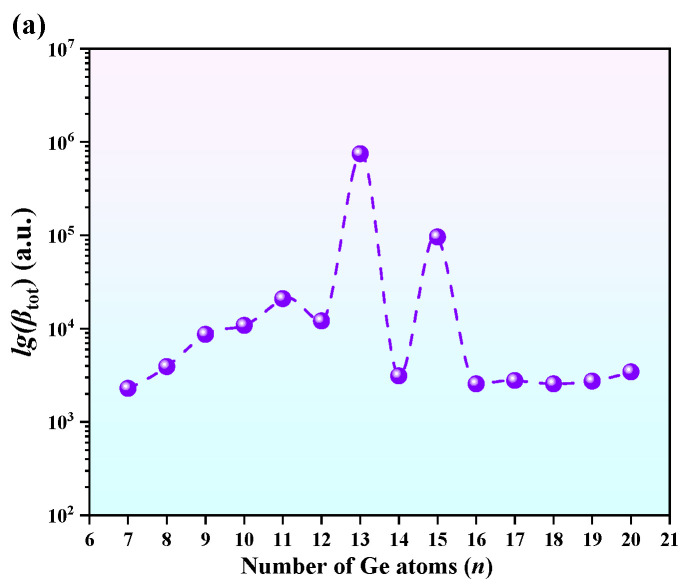

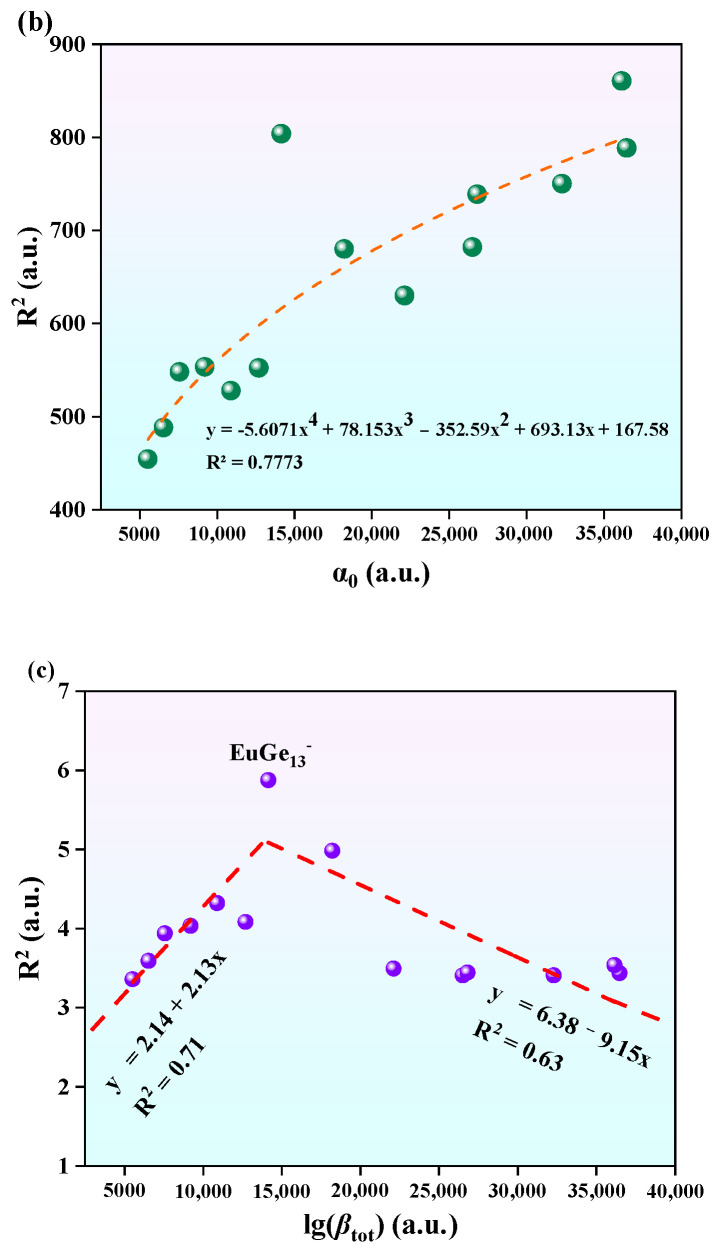
The relationship between (**a**) lg(*β*_tot)_ and cluster size and the relative relationships between (**b**) *α*_0_ and R^2^ and (**c**) lg(*β*_tot_) and R^2^ for different cluster sizes, with corresponding fitting curves.

To obtain a more comprehensive insight into the relationships among the parameters, we performed additional data processing and analysis. Figure 7a reflects the relationship between lg(*β*_tot_) and the cluster size. Figure 7b shows the relative relationship between *α*_0_ and R^2^, as well as the fitting curve. In Figure 7a, the hyperpolarizability of EuGe_13_^−^ is clearly higher than that of any other type. The second peak is observed for EuGe_15_^−^, which is consistent with the discussion above. In Figure 7b, *α*_0_ and R^2^ exhibit a better linear relationship, with a fitting R-squared value equal to 0.78. Finally, Figure 7c shows a volcano plot to further identify the best hyperpolarizability, which belongs to EuGe_13_^−^. Overall, the static polarizability exhibits a distinct positive correlation with R^2^, while the hyperpolarizability shows a trend of first increasing and then decreasing with R^2^. This suggests that as the electron cloud becomes more extended, the electrons become more responsive to external electric fields, thereby enhancing the likelihood of polarization. Furthermore, the volume and morphology of the clusters influence the outcome.

To avoid basis set effects, the hyperpolarizability was also calculated with aug-cc-pVTZ for Ge and ma-def2-TZVP for Eu [34]. The outcomes were consistent with the discussion above (seen Table S3, Supporting Information).

In summary, the nonlinear optical properties of EuGe*_n_*^−^ (*n* = 7–20) clusters are strongly correlated with their growth patterns and structural symmetry. As the cluster size increases, the (hyper)polarizability of the cluster exhibits a positive correlation with the overall electron space R^2^ and a negative correlation with the energy gap. The smaller *E*_g_ values might be the reason why the clusters have larger (hyper)polarizability values. Among the investigated clusters, EuGe_13_^−^ had the highest (hyper)polarizability value of 7.47 × 10^5^ a.u.; that is, R^2^ and *E*_g_ are two important descriptors for evaluating the nonlinear optical properties of EuGe*_n_*^−^ (*n* = 7–20).

### 2.6. Nonlinear Optical Properties of EuGe_13_^−^ from TD-DFT

**Figure 8:**
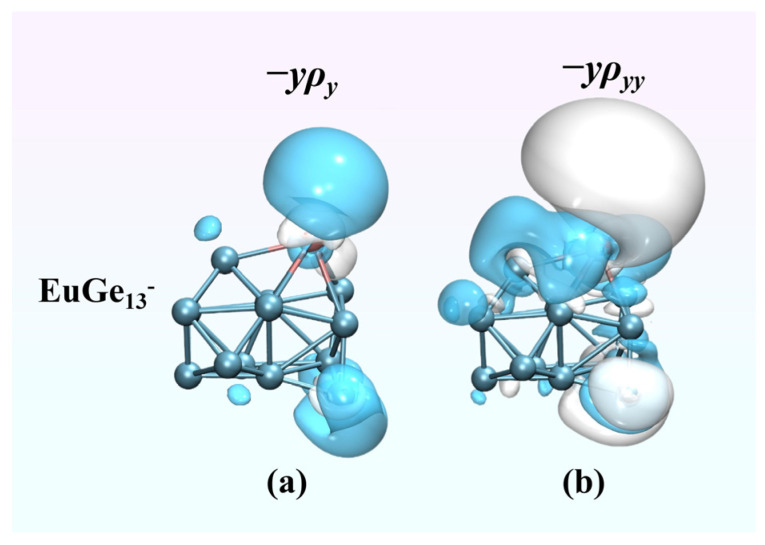
(**a**) The density of polarizability (isovalue = 0.6 a.u.) and (**b**) the density of hyperpolarizability (isovalue = 30 a.u.) of EuGe_13_^−^.

Whether compared within the same system or with some other materials [31,32,35], EuGe_13_^−^ displays significant *β*_tot_ values; thus, its (hyper)polarizability density was targeted for further examination. Figure 8 presents the local contributions of EuGe_13_^−^ polarizability and hyperpolarizability along the y direction in a static electric field. Positive contributions are represented by blue isosurfaces, whereas negative contributions are depicted by white isosurfaces. This visualization method facilitates a clearer understanding of the three-dimensional spatial contributions to the (hyper)polarizability. From Figure 8a, it is evident that the region of positive contribution substantially exceeds that of negative contribution. This discrepancy in areas provides a clear rationale for the cluster’s relatively large positive polarizability. For the hyperpolarizability, despite the presence of distinct positive and negative contribution areas in Figure 8b, the negative contribution regions are more extensive. This is consistent with the significantly negative hyperpolarizability of EuGe_13_^−^. In general, both the polarizability and hyperpolarizability exhibit a significant contribution region surrounding the Eu atom, which indicates that Eu atoms in the system have a stronger response to the external electric field, enhancing the nonlinear optical properties of the cluster.

**Figure 9:**
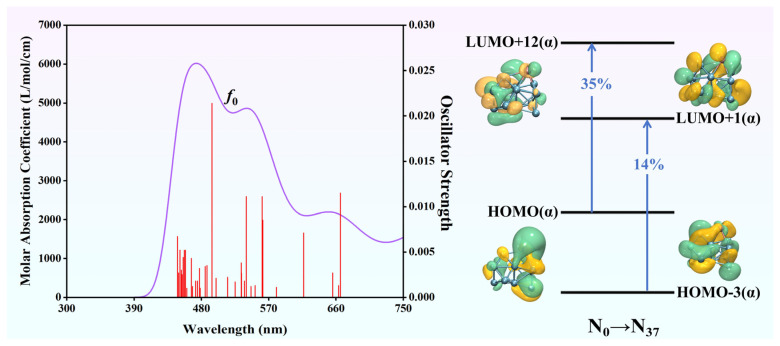
The simulated UV–vis spectrum and electronic excitation modes of the crucial excited state of EuGe_13_^−^.

The two-level expression (5) is widely employed to qualitatively investigate the NLO property of a system in depth [36,37]. Utilizing this formula, the crucial excitation energies of the EuGe_13_^−^ cluster were calculated within the framework of TD-DFT:

(5) $$\beta_{\mathrm{tot}}\propto \frac{\Delta \mu \cdot f_{0}}{\Delta E^{3}}$$

where Δ*μ* is the difference in dipole moment between the ground state and the crucial excited state, *f*_0_ is the oscillator strength, and Δ*E* is the transition energy of the key transition. Figure 9 shows the simulated UV–vis spectrum and the molecular orbital diagram of the main transitions of the EuGe_13_^−^ cluster. The strongest absorption peak at 2710.63 nm is dominated by N_0_ → N_1_, where N stands for a nonet. Δ*E* is 0.457a.u. The dominant electronic excitation mode in this transition is HOMO(α) → LUMO(α) with a contribution rate of 97%. N_0_ → N_37_ mainly contributes to the absorption peak at 474 nm. The *f*_0_ value of this crucial excitation is 0.0214 a.u., Δ*E* is 2.51 eV, and Δ*μ* is 0.348 a.u. Multiple electronic excitation modes are observed in this transition, with significant contributions from HOMO(α) → LUMO+12(α) (35%) and HOMO-3(α) → LUMO+1(α) (14%). The relatively large *β*_tot_ value of the cluster can be attributed to factors such as the large Δ*μ* and small Δ*E* values associated with the transitions. The relevant parameters for other clusters are collected in Table S4.

### 2.7. Density of States of EuGe_13_^−^

**Figure 10:**
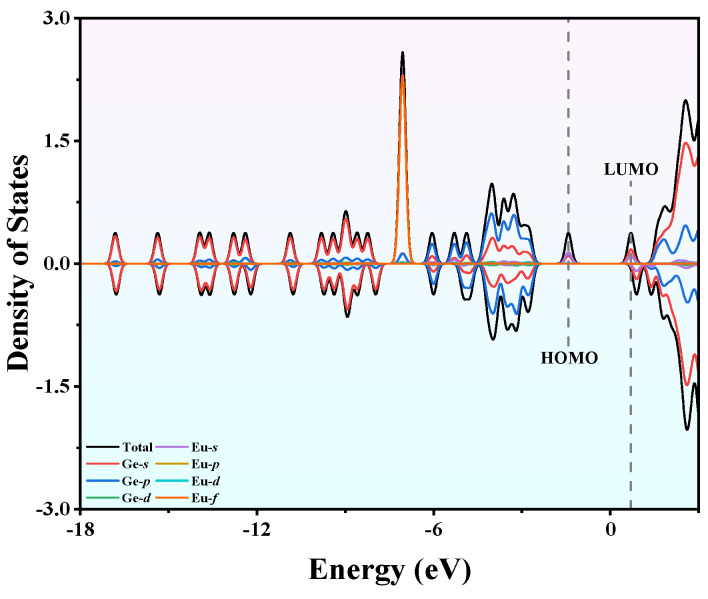
The DOS of the EuGe_13_^−^ cluster.

The density of states (DOSs) of a cluster can provide a deep understanding of the bonding characteristics within it. Figure 10 displays the DOS of the EuGe_13_^−^ cluster, where the black curve is the total DOS and the colored curves are the partial DOS curves of different orbits. As illustrated in the figure, the HOMO and LUMO are predominantly contributed by the Ge-*s*, *p*, and Eu-*s* orbitals; for the HOMO level, these contributions account for 25.91%, 35.13%, and 36.83%, respectively, and for the LUMO level, the corresponding contributions are 46.02%, 25.96%, and 27.11%. In the energy region below the HOMO, the most prominent peak in the DOS is observed at approximately −7.05 eV, which is predominantly attributed to the 4*f* orbitals of the Eu atom (89.18%). The remainder is mainly contributed by the Ge-*s* and *p* orbitals. It is evident that there is a pronounced asymmetry between the spin-up Alpha and spin-down Beta, especially the spin-up peak at −7.05 eV. Based on the NPA, it is once again demonstrated that the magnetic property of the cluster is mainly provided by the 4*f* orbitals of Eu and that the cluster exhibits spin polarization effects and magnetism.

## 3. Computational Details

All density functional theory calculations were performed using the Gaussian 09 quantum chemistry software [38]. The initial structures were obtained (1) using the ABCluster global searching software, version 3.1 and (2) from previously reported structures [39,40,41]. By way of (1), each cluster generated at least 400 isomers to ensure that the global minimum structure was found. The initial structures were optimized with TPSSh [42] combined with BS-1 basis sets (ECP28MWB [43] for Ge and ECP53MWB [44,45] for Eu). Then, the structures with an energy difference of within 0.8 eV were selected and further optimized using TPSSh combined with BS-2 basis sets (cc-pVTZ-PP [46,47] for Ge and ECP28MWB [48] for Eu). At the same time, frequency calculations were carried out to rule out imaginary frequencies, indicating that the structure is a true local minimum. Then, the above structures were further optimized at the mPW2PLYP [49] level with BS-2 basis sets, but frequency calculations were not carried out in order to reduce the calculation time. Finally, the single-point energy was calculated at the mPW2PLYP level with BS-3 basis sets (aug-cc-pVTZ [50] for Ge and ECP28MWB for Eu).

The PES spectra of EuGe*_n_*^−^ (*n* = 7–20) nanoclusters were obtained by applying Koopmans’ theorem [51,52] at the level of mPW2PLYP, and ultraviolet–visible (UV–vis) spectra were simulated via the time-dependent density functional theory method of the PBE0 functional with BS-3 basis sets [53,54]. The dipole moment, static polarizability, and first hyperpolarizability of each EuGe*_n_*^−^ (*n* = 7–20) cluster were calculated by means of the CAM-B3LYP functional [55] with BS-3 basis sets. The results were visualized using Multifwn [56,57] and VMD software, VMD 1.9.4 [58].

The formulas for the dipole moment (*μ*_0_), static polarizability (*α*_0_), and static first hyperpolarizability (*β*_tot_) are presented as follows:

(6) $$\mu_{0}={\left(\mu_{x}^{2}+\mu_{y}^{2}+\mu_{z}^{2}\right)}^{1/2}$$

(7) $$\alpha_{0}=1/3\left(\alpha_{xx}+\alpha_{yy}+\alpha_{zz}\right)$$

(8) $$\beta_{tot}={\left(\beta_{x}^{2}+\beta_{y}^{2}+\beta_{z}^{2}\right)}^{1/2}$$

where $\beta_{i}=\left(1/3\right)\sum_{j}\left(\beta_{ijj}^{2}+\beta_{jji}^{2}+\beta_{jij}^{2}\right)i,j=\left\{x,y,z\right\}$.

The ab initio molecular dynamics (AIMD) of EuGe*_n_*^−^ (*n* = 7–20) were obtained using Orca quantum chemistry code [59] at the level of the B97-3c functional [60] with def2-mTZVP for Ge and def2-mTZVP for Eu combined with Def2-ECP basis sets [48]. The B97-3c functional contains the Becke–Johnson damping scheme (D3BJ) [61,62].

mPW2PLYP displays high reliability in predicting the ground-state structures of rare-earth elements doped into silicon or germanium clusters. The different density functionals mPW2PLYP, TPSSh, PBE, wB97X, and B3LYP were compared with the ROCCSD(T) method for ScSi*_n_*^0/−^ (*n* = 4–9) in a previous work [63], and the results showed that all the ground-state structures predicted using mPW2PLYP were in line with those obtained using ROCCSD(T). Besides those of rare-earth-element-doped silicon clusters, the ground-state structures of ScGe*_n_*^−^ (*n* = 6–17) and CeGe*_n_*^−^ (*n* = 5–17) predicted using the mPW2PLYP approach agreed with those predicted via closed-shell DLPNO-CCSD(T1) and open-shell DLPNO-CCSD(T) methods [23,64], which further proved that the mPW2PLYP functional is suitable for such systems, combining accuracy and speed. The optimization scheme and TD-DFT calculation were also based on the above previous works.

## 4. Conclusions

This study systematically investigated the structural evolution and nonlinear optical properties of europium-doped germanium anionic clusters EuGe*_n_*^−^ (*n* = 7–20), utilizing an approach that combined global search techniques and double-hybrid density functional theory mPW2PLYP. The growth patterns were categorized into two distinct stages: (i) for *n* = 7–10, the structures were mainly characterized by an adsorption configuration with a Eu atom adsorbed onto a pure Ge cluster, and (ii) for *n* = 11–20, the clusters exhibited linked structures wherein a Eu atom was individually linked to a Ge_9_ sub-cluster with a TTP configuration and a Ge*_n_*_-9_ sub-cluster. The PES spectra, charge transfer, magnetic moments, relative stability, energy gaps, and (hyper)polarizability of the clusters were simulated and analyzed. EuGe_13_^−^, which exhibits notable NLO properties and a *β*_tot_ value of 7.47 × 10^5^ a.u., was subjected to a further investigation. The findings indicate that EuGe_13_^−^ holds potential as a nonlinear optical semiconductor material for application in multifunctional nanomaterials.

## Figures and Tables

**Table 1:** The NPA valence electron configuration, each valence orbital’s magnetic moment, the charge of the Eu atom in EuGe*_n_*^−^ (*n* = 7–20), and the total magnetic moment of the whole cluster.

Cluster	Charge (a.u.)	Electron Configuration	Magnetic Moment of Eu Atom					Molecule (μ*_B_*)
			6*s*	4*f*	5*d*	6*p*	Total	
EuGe_7_^−^	0.57	[core]6*s*^0.96^4*f*^6.98^5*d*^0.32^6*p*^0.18^	0.75	6.97	0.05	0.12	7.89	8
EuGe_8_^−^	0.51	[core]6*s*^0.89^4*f*^6.98^5*d*^0.40^6*p*^0.23^	0.71	6.97	0.05	0.13	7.86	8
EuGe_9_^−^	0.69	[core]6*s*^0.80^4*f*^6.98^5*d*^0.41^6*p*^0.14^	0.64	6.97	0.06	0.07	7.74	8
EuGe_10_^−^	0.67	[core]6*s*^1.00^4*f*^6.98^5*d*^0.18^6*p*^0.17^	0.70	6.98	0.03	0.13	7.84	8
EuGe_11_^−^	0.81	[core]6*s*^0.40^4*f*^6.97^5*d*^0.67^6*p*^0.17^	0.00	6.97	0.09	0.01	7.07	8
EuGe_12_^−^	0.80	[core]6*s*^0.41^4*f*^6.97^5*d*^0.69^6*p*^0.15^	0.01	6.97	0.13	0.01	7.12	8
EuGe_13_^−^	0.70	[core]6*s*^0.51^4*f*^6.97^5*d*^0.63^6*p*^0.21^	0.28	6.97	0.11	0.07	7.43	8
EuGe_14_^−^	0.69	[core]6*s*^0.27^4*f*^6.97^5*d*^0.79^6*p*^0.31^	0.01	6.97	0.09	0.01	7.08	8
EuGe_15_^−^	0.66	[core]6*s*^0.34^4*f*^6.97^5*d*^0.82^6*p*^0.24^	0.10	6.97	0.14	0.02	7.23	8
EuGe_16_^−^	0.74	[core]6*s*^0.27^4*f*^6.97^5*d*^0.75^6*p*^0.30^	0.01	6.97	0.09	0.00	7.07	8
EuGe_17_^−^	0.68	[core]6*s*^0.24^4*f*^6.97^5*d*^0.85^6*p*^0.29^	0.00	6.97	0.16	0.01	7.14	8
EuGe_18_^−^	0.73	[core]6*s*^0.24^4*f*^6.97^5*d*^0.82^6*p*^0.29^	0.04	6.96	0.12	0.01	7.13	8
EuGe_19_^−^	0.77	[core]6*s*^0.26^4*f*^6.97^5*d*^0.75^6*p*^0.28^	0.00	6.97	0.09	0.00	7.06	8
EuGe_20_^−^	0.66	[core]6*s*^0.26^4*f*^6.97^5*d*^0.83^6*p*^0.32^	0.02	6.96	0.09	0.01	7.08	8

**Table 2:** The dipole moment (*μ*_0_, in a.u.); static polarizability (*α*_0_, in a.u.); static first hyperpolarizability (*β*_tot_, in a.u.); components of hyperpolarizability in the x, y, and z directions (*β*_xxx_ *β*_yyy_ *β*_zzz_, in a.u.); and projection of hyperpolarizability in the direction of the dipole moment (*β*_prj_, in a.u.) of EuGe*_n_*^−^ (*n* = 7–20).

*n*.	*μ*_0_	*α*_0_	*β*_prj_	*β*_tot_	*β*_xxx_	*β*_yyy_	*β*_zzz_
7	1.37	454.46	−2242.88	2281.64	1179.93	1952.85	0.00
8	0.91	488.37	−3911.63	3911.63	0.00	0.00	−3911.63
9	2.02	548.01	8681.47	8681.48	107.48	8680.81	0.00
10	1.40	553.61	−10,775.99	10,801.04	−6283.05	−8785.54	0.00
11	3.94	527.99	−20,833.66	20,895.13	17,745.60	10,984.98	−1014.91
12	3.47	552.37	−12,056.43	12,066.61	5700.40	10,634.59	118.72
13	3.34	803.99	−726,411.06	747,032.61	373,853.70	−630,754.60	142,967.70
14	2.27	630.10	−2737.34	3113.67	1204.37	2871.31	0.00
15	3.48	680.11	−96,087.04	96,087.04	0.00	0.00	−96,087.04
16	2.98	682.23	−2359.59	2551.03	2538.74	−250.11	0.00
17	2.84	738.93	−2682.55	2776.80	137.43	−2771.25	−109.35
18	2.47	750.25	−2526.95	2557.41	−1881.68	−1731.94	0.00
19	2.70	788.58	−2690.12	2736.34	2618.65	793.88	0.00
20	2.53	860.61	−3405.02	3442.56	−3111.18	1473.71	0.00

## Data Availability

Data will be made available on request.

## Supplementary Materials

The following supporting information can be downloaded at https://www.mdpi.com/article/10.3390/molecules30061377/s1, Figure S1: The ab initio molecular dynamics of EuGe*_n_*^−^ (*n* = 7–20); Table S1: The Cartesian coordinates of EuGe*_n_*^−^ (*n* = 7–20); Table S2: Total energies at mPW2PLYP with zero-point energy at TPSSh (in Hartree); Table S3: The dipole moment (*μ*_0_, in a.u.), static polarizability (*α*_0_ in a.u.), static first hyperpolarizability (*β*_tot_, in a.u.), the components of hyperpolarizability in the x, y, and z directions (*β*_xxx_ *β*_yyy_ *β*_zzz_, in a.u.) as well as the projection of the hyperpolarizability in the direction of the dipole moment (*β*_prj_, in a.u.) of EuGe*_n_*^−^ (*n* = 7–20) with BS4 (aug-cc-pVTZ for Ge and ma-def2-TZVP for Eu); Table S4: The main transitions, oscillator strengths (*f*_0_, in a.u.), difference in dipole moment (Δ*μ*, in a.u.) transition energies (Δ*E*, in eV), wavelengths (λ, in nm) and orbital contribution of the crucial excited states for the EuGe*_n_*^−^ (*n* = 7–20) cluster.
