# Supplementary material for: Structural, Electronic, and Nonlinear Optical Characteristics of Europium-Doped Germanium Anion Nanocluster EuGen− (n = 7–20): A Theoretical Investigation
Source: Molecules. 2025 Mar 19;30(6):1377. doi: 10.3390/molecules30061377 (PMC11946336; doi:10.3390/molecules30061377)
Supplement: Supplementary file 1 [file molecules-30-01377-s001.zip › molecules-3524060-supplementary.pdf]

# Supplementary Information

Structural, Electronic, and Nonlinear Optical Characteristics of Europium-Doped Germanium Anion Nanocluster $EuGe_n^-$ ($n$ = 7–20): A Theoretical Investigation

**Chenliang Hao [1], Xueyan Dong [1], Chunli Li [2], Caixia Dong [2], Zhaofeng Yang [2,*] and Jucai Yang [1,2]**

[1] Inner Mongolia Key Laboratory of Theoretical and Computational Chemistry Simulation, School of Chemical Engineering, Inner Mongolia University of Technology, Hohhot 010051, China
[2] School of Resources and Environmental Engineering, Inner Mongolia University of Technology, Hohhot 010051, China;
* Correspondence: yangzf@imut.edu.cn

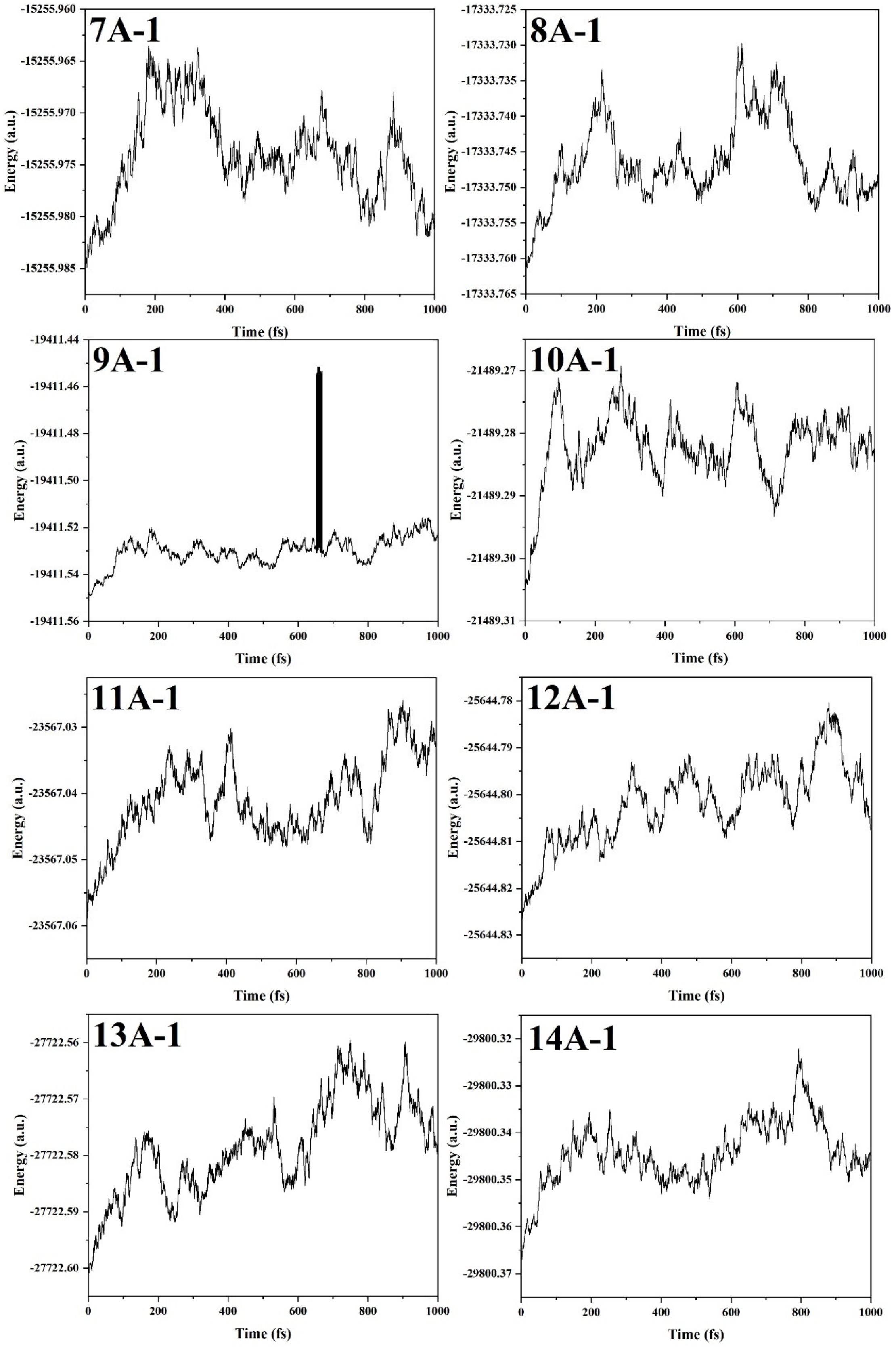
7A-1
8A-1
9A-1
10A-1
11A-1
12A-1
13A-1
14A-1
Energy (a.u.)
Time (fs)

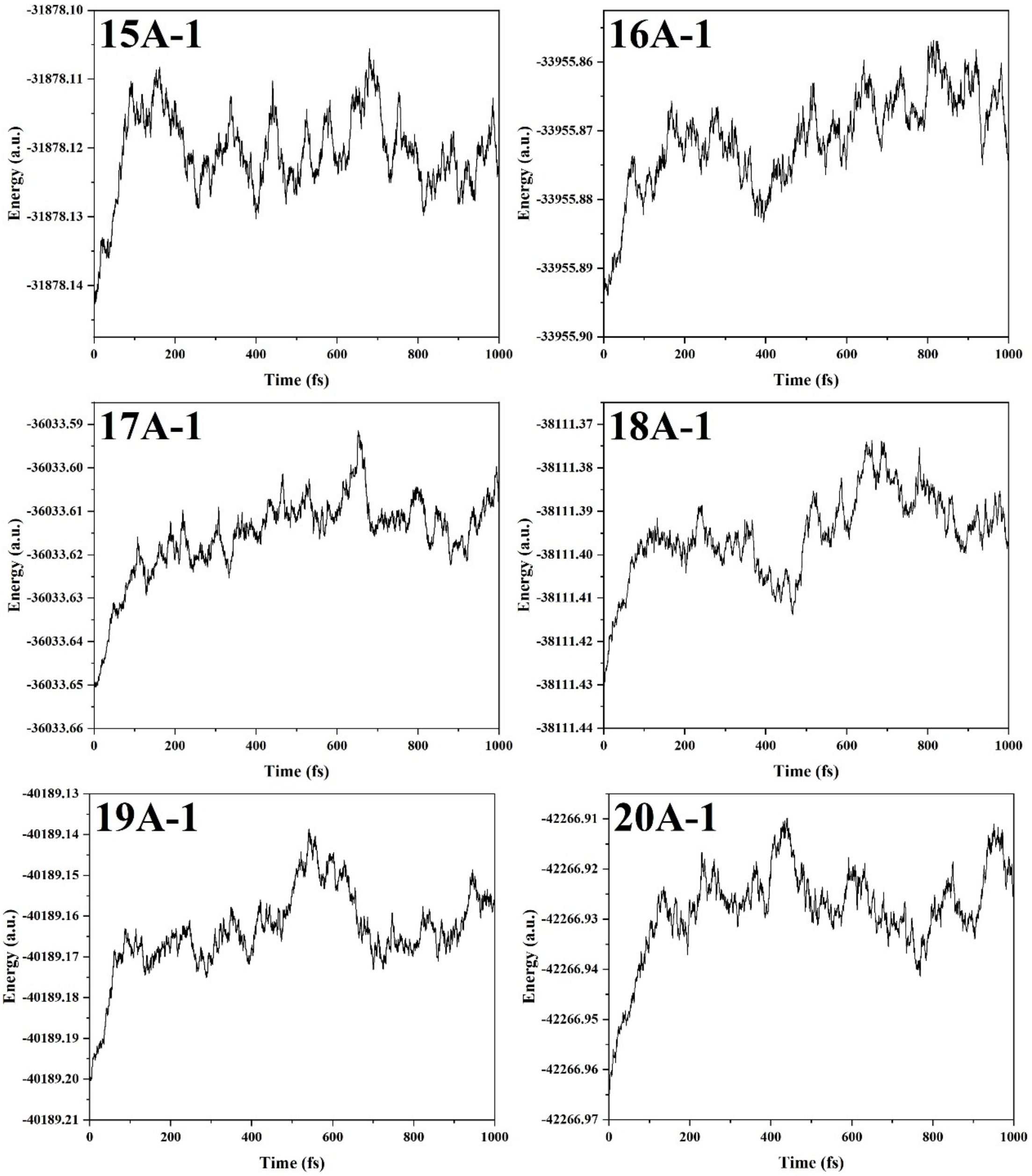


Figure S1. The *ab initio* molecular dynamics of $EuGe_n^-$ ($n$ = 7–20).

Table S1. The Cartesian coordinates of $EuGe_n^-$ ($n$ = 7–20).

**7A-1**

| | | | | |
|---|---|---|---|---|
| 63 | 0 | −2.098269 | −2.198227 | 0.000000 |
| 32 | 0 | 0.566316 | −1.121861 | 1.294346 |
| 32 | 0 | 2.302892 | 0.563950 | 0.000000 |
| 32 | 0 | 0.656105 | 2.754410 | 0.000000 |
| 32 | 0 | −1.093294 | 0.694793 | 0.000000 |
| 32 | 0 | 0.566316 | 1.279164 | 2.094889 |
| 32 | 0 | 0.566316 | −1.121861 | −1.294346 |
| 32 | 0 | 0.566316 | 1.279164 | −2.094889 |

**7A-2**

| | | | | |
|---|---|---|---|---|
| 63 | 0 | 2.119759 | 0.043440 | 0.000000 |
| 32 | 0 | −1.549132 | 0.400420 | 0.000000 |
| 32 | 0 | −0.108673 | 1.049930 | 2.122484 |
| 32 | 0 | −0.108673 | −1.493246 | 1.347618 |
| 32 | 0 | 0.032227 | 2.477089 | 0.000000 |
| 32 | 0 | −2.221680 | −2.076400 | 0.000000 |
| 32 | 0 | −0.108673 | 1.049930 | −2.122484 |
| 32 | 0 | −0.108673 | −1.493246 | −1.347618 |

**7A-3**

| | | | | |
|---|---|---|---|---|
| 63 | 0 | 2.183520 | −0.211019 | 0.000000 |
| 32 | 0 | 1.216001 | 2.732871 | 0.000000 |
| 32 | 0 | −0.283080 | 1.380555 | 1.419869 |
| 32 | 0 | −2.063021 | 0.169573 | 0.000000 |
| 32 | 0 | −2.319465 | −2.305219 | 0.000000 |
| 32 | 0 | −0.283080 | −1.471446 | 1.325521 |
| 32 | 0 | −0.283080 | 1.380555 | −1.419869 |
| 32 | 0 | −0.283080 | −1.471446 | −1.325521 |

**8A-1**

| | | | | |
|---|---|---|---|---|
| 63 | 0 | 0.000000 | 0.000000 | 3.059011 |
| 32 | 0 | 0.000000 | 1.509507 | 0.297783 |
| 32 | 0 | 0.000000 | 2.055839 | −2.173189 |
| 32 | 0 | −2.072254 | 0.000000 | 0.677626 |
| 32 | 0 | −1.450544 | 0.000000 | −1.813434 |
| 32 | 0 | 2.072254 | 0.000000 | 0.677626 |
| 32 | 0 | 1.450544 | 0.000000 | −1.813434 |
| 32 | 0 | 0.000000 | −1.509507 | 0.297783 |
| 32 | 0 | 0.000000 | −2.055839 | −2.173189 |

**8A-2**

| | | | | |
|---|---|---|---|---|
| 63 | 0 | 2.365775 | 0.474586 | 0.000000 |

| | | | | |
|---|---|---|---|---|
| 32 | 0 | −0. 508617 | 1. 918358 | 0. 000000 |
| 32 | 0 | −1. 893892 | 0. 087370 | 1. 516521 |
| 32 | 0 | 0. 330090 | −1. 406356 | 1. 447897 |
| 32 | 0 | 0. 330090 | 0. 983148 | 2. 299877 |
| 32 | 0 | −1. 681580 | −2. 181021 | 0. 000000 |
| 32 | 0 | −1. 893892 | 0. 087370 | −1. 516521 |
| 32 | 0 | 0. 330090 | −1. 406356 | −1. 447897 |
| 32 | 0 | 0. 330090 | 0. 983148 | −2. 299877 |

**8A-3**

| | | | | |
|---|---|---|---|---|
| 63 | 0 | 2. 198251 | −0. 062976 | 0. 000000 |
| 32 | 0 | 0. 571186 | −2. 762992 | 0. 000000 |
| 32 | 0 | −2. 156496 | −2. 136392 | 0. 000000 |
| 32 | 0 | −0. 433334 | 1. 753212 | 1. 352748 |
| 32 | 0 | −2. 352621 | 0. 637941 | 0. 000000 |
| 32 | 0 | −0. 433334 | −0. 989209 | 1. 484727 |
| 32 | 0 | 1. 343458 | 2. 857420 | 0. 000000 |
| 32 | 0 | −0. 433334 | 1. 753212 | −1. 352748 |
| 32 | 0 | −0. 433334 | −0. 989209 | −1. 484727 |

**9A-1**

| | | | | |
|---|---|---|---|---|
| 63 | 0 | −0. 035436 | 3. 211647 | 0. 000000 |
| 32 | 0 | −1. 568936 | −2. 092119 | 0. 000000 |
| 32 | 0 | −1. 268410 | −0. 680963 | −2. 210606 |
| 32 | 0 | 0. 814784 | 0. 646847 | −1. 417968 |
| 32 | 0 | −1. 642711 | 0. 620529 | 0. 000000 |
| 32 | 0 | 0. 814784 | −2. 068513 | −1. 377032 |
| 32 | 0 | 2. 559098 | −0. 646082 | 0. 000000 |
| 32 | 0 | −1. 268410 | −0. 680963 | 2. 210606 |
| 32 | 0 | 0. 814784 | 0. 646847 | 1. 417968 |
| 32 | 0 | 0. 814784 | −2. 068513 | 1. 377032 |

**9A-2**

| | | | | |
|---|---|---|---|---|
| 63 | 0 | −1. 425405 | −2. 651180 | 0. 000000 |
| 32 | 0 | 0. 538821 | 2. 431665 | 0. 000000 |
| 32 | 0 | 2. 464920 | 0. 604143 | 0. 000000 |
| 32 | 0 | 1. 046606 | 1. 175784 | 2. 239409 |
| 32 | 0 | 1. 046606 | −1. 233935 | 1. 335386 |
| 32 | 0 | −1. 221911 | 0. 124468 | 1. 418209 |
| 32 | 0 | −1. 940075 | 2. 051069 | 0. 000000 |
| 32 | 0 | 1. 046606 | 1. 175784 | −2. 239409 |
| 32 | 0 | 1. 046606 | −1. 233935 | −1. 335386 |
| 32 | 0 | −1. 221911 | 0. 124468 | −1. 418209 |

**9A-3**

| | | | | |
|---|---|---|---|---|
| 63 | 0 | 2.635541 | 0.000206 | 0.000001 |
| 32 | 0 | -0.093728 | -0.013274 | -1.851230 |
| 32 | 0 | 0.509888 | -2.331752 | -1.324089 |
| 32 | 0 | -2.145661 | 0.011316 | 1.592221 |
| 32 | 0 | 0.509770 | 0.019403 | 2.681015 |
| 32 | 0 | 0.509489 | 2.312641 | -1.357276 |
| 32 | 0 | -2.146179 | 1.373130 | -0.805591 |
| 32 | 0 | -2.145811 | -1.384719 | -0.785923 |
| 32 | 0 | -0.093376 | 1.609921 | 0.913850 |
| 32 | 0 | -0.093113 | -1.597074 | 0.937023 |

**10A-1**

| | | | | |
|---|---|---|---|---|
| 63 | 0 | 2.374766 | 2.778705 | 0.000000 |
| 32 | 0 | -1.973628 | 1.794568 | 0.000000 |
| 32 | 0 | -1.551806 | -2.475271 | 0.000000 |
| 32 | 0 | 1.762494 | -0.371302 | 0.000000 |
| 32 | 0 | 0.116795 | 1.058421 | 1.435780 |
| 32 | 0 | 1.030798 | -2.857225 | 0.000000 |
| 32 | 0 | 0.116795 | -1.429220 | 2.044136 |
| 32 | 0 | -2.205178 | -0.409874 | 1.421376 |
| 32 | 0 | 0.116795 | 1.058421 | -1.435780 |
| 32 | 0 | 0.116795 | -1.429220 | -2.044136 |
| 32 | 0 | -2.205178 | -0.409874 | -1.421376 |

**10A-2**

| | | | | |
|---|---|---|---|---|
| 63 | 0 | 3.679095 | 0.838475 | 0.000000 |
| 32 | 0 | 1.011196 | -0.141196 | 1.424645 |
| 32 | 0 | 0.350936 | -2.188576 | 0.000000 |
| 32 | 0 | -2.288617 | -2.392287 | 0.000000 |
| 32 | 0 | -2.904138 | 0.158910 | 0.000000 |
| 32 | 0 | -1.291247 | 1.513558 | 1.472624 |
| 32 | 0 | -1.291247 | -1.030494 | 2.024953 |
| 32 | 0 | 0.741193 | 2.087470 | 0.000000 |
| 32 | 0 | 1.011196 | -0.141196 | -1.424645 |
| 32 | 0 | -1.291247 | 1.513558 | -1.472624 |
| 32 | 0 | -1.291247 | -1.030494 | -2.024953 |

**10A-3**

| | | | | |
|---|---|---|---|---|
| 63 | 0 | -0.533396 | -3.282399 | 0.000000 |
| 32 | 0 | 2.388989 | -1.702956 | 0.000000 |
| 32 | 0 | -1.337016 | 0.832166 | 2.224983 |
| 32 | 0 | 0.620724 | -0.504558 | 1.384010 |
| 32 | 0 | 2.442522 | 0.973998 | 0.000000 |

| | | | | |
|---|---|---|---|---|
| 32 | 0 | −1.735869 | 2.229202 | 0.000000 |
| 32 | 0 | −1.854379 | −0.476331 | 0.000000 |
| 32 | 0 | 0.620724 | 2.391548 | 1.341053 |
| 32 | 0 | −1.337016 | 0.832166 | −2.224983 |
| 32 | 0 | 0.620724 | −0.504558 | −1.384010 |
| 32 | 0 | 0.620724 | 2.391548 | −1.341053 |

**11A-1**

| | | | | |
|---|---|---|---|---|
| 63 | 0 | −2.914394 | −1.323351 | 0.060998 |
| 32 | 0 | −0.680898 | 0.642306 | −1.120628 |
| 32 | 0 | −1.785317 | 1.546072 | 1.345469 |
| 32 | 0 | 1.814346 | −0.576813 | −2.069377 |
| 32 | 0 | −0.193807 | −0.798114 | 1.416452 |
| 32 | 0 | 2.187175 | −1.952434 | 0.258929 |
| 32 | 0 | 2.079168 | −0.148977 | 2.268740 |
| 32 | 0 | −3.078550 | 1.632638 | −0.714705 |
| 32 | 0 | 0.842481 | 1.727277 | 1.043647 |
| 32 | 0 | 3.221115 | 0.475802 | −0.106544 |
| 32 | 0 | −0.119264 | −1.930085 | −0.982149 |
| 32 | 0 | 1.451264 | 1.987674 | −1.459924 |

**11A-2**

| | | | | |
|---|---|---|---|---|
| 63 | 0 | −2.555637 | −1.555166 | −0.236269 |
| 32 | 0 | −0.461502 | 0.396859 | 1.277473 |
| 32 | 0 | 0.423439 | −1.976793 | 0.339499 |
| 32 | 0 | 2.937599 | −0.840115 | 0.300420 |
| 32 | 0 | 1.489994 | −0.790946 | 2.443400 |
| 32 | 0 | 2.015660 | 1.547617 | 1.259822 |
| 32 | 0 | −2.933034 | 1.032531 | 1.338848 |
| 32 | 0 | 0.180638 | 2.396346 | −0.457685 |
| 32 | 0 | −0.243964 | 0.049322 | −1.504536 |
| 32 | 0 | 1.755876 | −1.423572 | −1.971701 |
| 32 | 0 | 2.311537 | 1.126614 | −1.463438 |
| 32 | 0 | −2.444834 | 1.543869 | −1.096948 |

**11A-3**

| | | | | |
|---|---|---|---|---|
| 63 | 0 | 0.000000 | 0.000000 | 1.882914 |
| 32 | 0 | −1.367059 | 2.094990 | −0.193149 |
| 32 | 0 | 0.000000 | 4.445419 | −0.274195 |
| 32 | 0 | 0.000000 | 2.909850 | −2.270722 |
| 32 | 0 | 0.000000 | 0.000000 | −1.446110 |
| 32 | 0 | 0.000000 | 3.072670 | 1.800778 |
| 32 | 0 | −1.367059 | −2.094990 | −0.193149 |
| 32 | 0 | 0.000000 | −4.445419 | −0.274195 |

| | | | | |
|---|---|---|---|---|
| 32 | 0 | 0.000000 | -2.909850 | -2.270722 |
| 32 | 0 | 0.000000 | -3.072670 | 1.800778 |
| 32 | 0 | 1.367059 | -2.094990 | -0.193149 |
| 32 | 0 | 1.367059 | 2.094990 | -0.193149 |

**11A-4**

| | | | | |
|---|---|---|---|---|
| 63 | 0 | -2.869060 | 2.414607 | 0.000000 |
| 32 | 0 | 0.252131 | 3.016536 | 0.000000 |
| 32 | 0 | -0.023954 | -1.606602 | 2.074510 |
| 32 | 0 | -0.023954 | 0.875790 | 1.373587 |
| 32 | 0 | 2.358577 | -0.798460 | 1.329979 |
| 32 | 0 | -1.778058 | -0.504901 | 0.000000 |
| 32 | 0 | 1.382115 | -2.839668 | 0.000000 |
| 32 | 0 | 2.397512 | 1.557921 | 0.000000 |
| 32 | 0 | -1.226574 | -2.925100 | 0.000000 |
| 32 | 0 | -0.023954 | -1.606602 | -2.074510 |
| 32 | 0 | -0.023954 | 0.875790 | -1.373587 |
| 32 | 0 | 2.358577 | -0.798460 | -1.329979 |

**12A-1**

| | | | | |
|---|---|---|---|---|
| 63 | 0 | 2.164093 | 2.171931 | -0.010437 |
| 32 | 0 | -0.280086 | -2.242218 | -0.505605 |
| 32 | 0 | 0.130945 | 0.105422 | -1.468133 |
| 32 | 0 | 2.889682 | -0.470354 | 1.319060 |
| 32 | 0 | 2.380671 | -2.583889 | 0.081471 |
| 32 | 0 | -1.712679 | 0.715638 | 2.425968 |
| 32 | 0 | -2.150482 | -1.649803 | 1.290091 |
| 32 | 0 | -3.173291 | 0.637912 | 0.276892 |
| 32 | 0 | 0.294082 | -0.323435 | 1.192872 |
| 32 | 0 | -2.526401 | -1.292903 | -1.440221 |
| 32 | 0 | 2.760696 | -0.523421 | -1.409626 |
| 32 | 0 | -2.004144 | 1.317803 | -2.013569 |
| 32 | 0 | -0.869552 | 2.033260 | 0.271349 |

**12A-2**

| | | | | |
|---|---|---|---|---|
| 63 | 0 | 0.000000 | -0.000000 | 1.320008 |
| 32 | 0 | -2.393314 | -4.270110 | 0.327675 |
| 32 | 0 | -0.027770 | 1.404783 | -1.704964 |
| 32 | 0 | -1.842436 | -2.500053 | 2.063533 |
| 32 | 0 | -2.244397 | -1.666643 | -0.366882 |
| 32 | 0 | 1.367480 | 3.656799 | -1.931026 |
| 32 | 0 | 0.000000 | 3.070862 | 0.312281 |
| 32 | 0 | 2.393314 | 4.270110 | 0.327675 |
| 32 | 0 | 0.027770 | -1.404783 | -1.704964 |

| | | | | |
|---|---|---|---|---|
| 32 | 0 | 1.842436 | 2.500053 | 2.063533 |
| 32 | 0 | 2.244397 | 1.666643 | -0.366882 |
| 32 | 0 | -1.367480 | -3.656799 | -1.931026 |
| 32 | 0 | -0.000000 | -3.070862 | 0.312281 |

**13A-1**

| | | | | |
|---|---|---|---|---|
| 63 | 0 | -2.129602 | 2.509847 | -0.396222 |
| 32 | 0 | 0.934225 | 2.480883 | 0.420856 |
| 32 | 0 | -1.643369 | -2.964990 | -1.280420 |
| 32 | 0 | 3.215425 | -1.108091 | 0.241085 |
| 32 | 0 | -2.896796 | 0.100328 | 1.459255 |
| 32 | 0 | -1.878817 | -2.186715 | 1.123867 |
| 32 | 0 | -2.418089 | -0.586779 | -1.011535 |
| 32 | 0 | 0.109188 | 0.834196 | -1.565956 |
| 32 | 0 | 2.108362 | -0.598836 | -2.287933 |
| 32 | 0 | 2.884498 | 1.304039 | -0.704017 |
| 32 | 0 | 0.536627 | -1.739230 | -0.630037 |
| 32 | 0 | 2.466469 | 0.758393 | 1.892531 |
| 32 | 0 | -0.303556 | 0.388532 | 1.239294 |
| 32 | 0 | 1.078487 | -1.622990 | 1.883072 |

**13A-2**

| | | | | |
|---|---|---|---|---|
| 63 | 0 | 2.594265 | 3.025812 | 0.000000 |
| 32 | 0 | -2.645204 | -0.875513 | 0.000000 |
| 32 | 0 | 0.381800 | -3.130237 | 2.103660 |
| 32 | 0 | 0.381800 | 1.496436 | 2.100439 |
| 32 | 0 | 1.669160 | -2.587750 | 0.000000 |
| 32 | 0 | -2.302365 | 1.438163 | 1.372448 |
| 32 | 0 | 1.486682 | 0.150128 | 0.000000 |
| 32 | 0 | -0.527916 | -0.782318 | 1.480468 |
| 32 | 0 | -0.951369 | -3.398611 | 0.000000 |
| 32 | 0 | -0.533364 | 2.710591 | 0.000000 |
| 32 | 0 | 0.381800 | -3.130237 | -2.103660 |
| 32 | 0 | 0.381800 | 1.496436 | -2.100439 |
| 32 | 0 | -2.302365 | 1.438163 | -1.372448 |
| 32 | 0 | -0.527916 | -0.782318 | -1.480468 |

**13A-3**

| | | | | |
|---|---|---|---|---|
| 63 | 0 | 2.405967 | -1.297038 | 0.000000 |
| 32 | 0 | -1.788669 | 3.264490 | 0.000000 |
| 32 | 0 | -0.704806 | 2.073544 | 2.127586 |
| 32 | 0 | -2.178667 | -2.378504 | 0.000000 |
| 32 | 0 | 0.205458 | -3.585918 | 0.000000 |
| 32 | 0 | -0.754781 | -2.622171 | 2.138762 |

| | | | | |
|---|---|---|---|---|
| 32 | 0 | -0.153805 | -0.264782 | 1.316734 |
| 32 | 0 | -2.198272 | 0.674168 | 0.000000 |
| 32 | 0 | 1.847263 | 1.471694 | 1.426455 |
| 32 | 0 | 0.755661 | 3.262737 | 0.000000 |
| 32 | 0 | -0.704806 | 2.073544 | -2.127586 |
| 32 | 0 | -0.754781 | -2.622171 | -2.138762 |
| 32 | 0 | -0.153805 | -0.264782 | -1.316734 |
| 32 | 0 | 1.847263 | 1.471694 | -1.426455 |

**14A-1**

| | | | | |
|---|---|---|---|---|
| 32 | 0 | -3.580008 | -1.012178 | 1.467910 |
| 32 | 0 | -3.234327 | -3.182302 | 0.000000 |
| 32 | 0 | -1.275756 | -2.536546 | 1.625640 |
| 32 | 0 | -2.707382 | 0.980092 | 0.000000 |
| 32 | 0 | -1.275756 | 0.031578 | 2.107409 |
| 32 | 0 | -0.077411 | -0.919969 | 0.000000 |
| 32 | 0 | 2.317379 | -0.253546 | 0.000000 |
| 63 | 0 | 0.160436 | 2.205522 | 0.000000 |
| 32 | 0 | 4.857350 | 2.606844 | 0.000000 |
| 32 | 0 | 4.756437 | 0.047509 | 0.000000 |
| 32 | 0 | 3.017567 | 1.706771 | 1.429400 |
| 32 | 0 | -3.580008 | -1.012178 | -1.467910 |
| 32 | 0 | -1.275756 | -2.536546 | -1.625640 |
| 32 | 0 | -1.275756 | 0.031578 | -2.107409 |
| 32 | 0 | 3.017567 | 1.706771 | -1.429400 |

**14A-2**

| | | | | |
|---|---|---|---|---|
| 63 | 0 | -2.867090 | -0.737177 | 0.000000 |
| 32 | 0 | 2.321138 | 2.479578 | -0.000000 |
| 32 | 0 | -0.771161 | -2.649673 | 1.394834 |
| 32 | 0 | -0.100839 | -0.145583 | 1.322255 |
| 32 | 0 | 2.170545 | -0.187847 | -0.000000 |
| 32 | 0 | -0.109805 | 3.476389 | -0.000000 |
| 32 | 0 | 1.456082 | -3.140840 | 0.000000 |
| 32 | 0 | 0.843361 | 2.149731 | 2.180020 |
| 32 | 0 | 1.613345 | -1.836598 | 2.240190 |
| 32 | 0 | -1.681394 | 1.894141 | 1.374193 |
| 32 | 0 | -0.771161 | -2.649673 | -1.394834 |
| 32 | 0 | -0.100839 | -0.145583 | -1.322255 |
| 32 | 0 | 0.843361 | 2.149731 | -2.180020 |
| 32 | 0 | 1.613345 | -1.836598 | -2.240190 |
| 32 | 0 | -1.681394 | 1.894141 | -1.374193 |

**14A-3**

| | | | | |
|---|---|---|---|---|
| 63 | 0 | 2.286548 | −2.142669 | 0.000000 |
| 32 | 0 | −0.639705 | −3.224075 | 0.000000 |
| 32 | 0 | 0.059923 | −2.242807 | −2.299233 |
| 32 | 0 | −2.281711 | −1.630612 | −1.406276 |
| 32 | 0 | −1.141343 | 2.082522 | −2.174952 |
| 32 | 0 | −0.819522 | 3.552221 | 0.000000 |
| 32 | 0 | 1.329216 | 2.765261 | −1.366150 |
| 32 | 0 | 2.445226 | 0.916327 | 0.000000 |
| 32 | 0 | 0.280222 | 0.113647 | −1.362034 |
| 32 | 0 | −1.980255 | 0.797887 | 0.000000 |
| 32 | 0 | 0.059923 | −2.242807 | 2.299233 |
| 32 | 0 | −2.281711 | −1.630612 | 1.406276 |
| 32 | 0 | −1.141343 | 2.082522 | 2.174952 |
| 32 | 0 | 1.329216 | 2.765261 | 1.366150 |
| 32 | 0 | 0.280222 | 0.113647 | 1.362034 |

**15A-1**

| | | | | |
|---|---|---|---|---|
| 63 | 0 | 0.000000 | 0.000000 | 3.062779 |
| 32 | 0 | 0.000000 | 2.540037 | −2.615771 |
| 32 | 0 | −2.141613 | 2.152811 | −1.084422 |
| 32 | 0 | −1.304119 | 0.000000 | 0.253216 |
| 32 | 0 | −1.352269 | 2.384452 | 1.421695 |
| 32 | 0 | 0.000000 | 3.791348 | −0.344516 |
| 32 | 0 | 0.000000 | 0.000000 | −1.964798 |
| 32 | 0 | 0.000000 | −2.540037 | −2.615771 |
| 32 | 0 | −2.141613 | −2.152811 | −1.084422 |
| 32 | 0 | −1.352269 | −2.384452 | 1.421695 |
| 32 | 0 | 0.000000 | −3.791348 | −0.344516 |
| 32 | 0 | 2.141613 | −2.152811 | −1.084422 |
| 32 | 0 | 1.304119 | 0.000000 | 0.253216 |
| 32 | 0 | 1.352269 | −2.384452 | 1.421695 |
| 32 | 0 | 2.141613 | 2.152811 | −1.084422 |
| 32 | 0 | 1.352269 | 2.384452 | 1.421695 |

**15A-2**

| | | | | |
|---|---|---|---|---|
| 63 | 0 | −1.212822 | 2.037801 | −0.146591 |
| 32 | 0 | 1.583873 | −1.933326 | 0.360611 |
| 32 | 0 | −4.377733 | 1.596601 | 0.240332 |
| 32 | 0 | 2.809226 | −0.946474 | 2.398982 |
| 32 | 0 | 2.724185 | −1.689696 | −1.925017 |
| 32 | 0 | −1.027136 | −1.161168 | −0.161941 |
| 32 | 0 | 0.999104 | 0.551034 | 1.385169 |
| 32 | 0 | −5.369640 | −0.753899 | 0.127451 |

| | | | | |
|---|---|---|---|---|
| 32 | 0 | -3.276628 | -0.113010 | -1.372447 |
| 32 | 0 | 3.685734 | 1.201729 | 1.180156 |
| 32 | 0 | -3.037275 | -0.293625 | 1.377976 |
| 32 | 0 | 4.336848 | -1.196122 | 0.170282 |
| 32 | 0 | 1.969821 | 2.298497 | -0.398808 |
| 32 | 0 | 3.792389 | 0.743710 | -1.536860 |
| 32 | 0 | 1.036309 | 0.115353 | -1.424395 |
| 32 | 0 | -3.461334 | -2.431526 | -0.132891 |

**15A-3**

| | | | | |
|---|---|---|---|---|
| 63 | 0 | 1.260720 | -1.989911 | -1.052768 |
| 32 | 0 | 0.760837 | 3.252436 | -0.413275 |
| 32 | 0 | -3.643592 | 1.259565 | 1.094277 |
| 32 | 0 | 2.326860 | -0.261000 | 1.983785 |
| 32 | 0 | 3.850543 | -1.917698 | 0.795190 |
| 32 | 0 | -0.334254 | 0.562208 | -1.043372 |
| 32 | 0 | -1.942550 | -1.167500 | -2.102468 |
| 32 | 0 | -1.573636 | -2.396646 | 0.275087 |
| 32 | 0 | -0.102604 | -0.479814 | 1.315498 |
| 32 | 0 | -2.765191 | 1.338955 | -1.418886 |
| 32 | 0 | -3.760653 | -0.990073 | -0.264015 |
| 32 | 0 | 3.741137 | 0.241267 | -0.420394 |
| 32 | 0 | -1.153500 | 1.855857 | 1.049933 |
| 32 | 0 | 2.659995 | 2.051997 | 0.837646 |
| 32 | 0 | -2.452363 | -0.876353 | 2.229856 |
| 32 | 0 | 1.906928 | 1.444437 | -1.846225 |

**16A-1**

| | | | | |
|---|---|---|---|---|
| 63 | 0 | -2.416991 | 0.228615 | 0.000000 |
| 32 | 0 | 0.652330 | 1.825095 | 0.000000 |
| 32 | 0 | -1.366457 | 2.975531 | 1.309742 |
| 32 | 0 | 0.995011 | 3.419901 | 2.111127 |
| 32 | 0 | 2.429327 | 3.807921 | 0.000000 |
| 32 | 0 | -0.054901 | 4.968052 | 0.000000 |
| 32 | 0 | 0.525128 | -0.677549 | 0.000000 |
| 32 | 0 | -0.675370 | -1.646576 | 2.108220 |
| 32 | 0 | -1.932560 | -2.825333 | 0.000000 |
| 32 | 0 | 1.817746 | -2.236871 | 1.599056 |
| 32 | 0 | 1.989389 | -4.308347 | 0.000000 |
| 32 | 0 | -0.196061 | -4.131947 | 1.468040 |
| 32 | 0 | -1.366457 | 2.975531 | -1.309742 |
| 32 | 0 | 0.995011 | 3.419901 | -2.111127 |
| 32 | 0 | -0.675370 | -1.646576 | -2.108220 |
| 32 | 0 | 1.817746 | -2.236871 | -1.599056 |

| | | | | | |
|---|---|---|---|---|---|
| | 32 | 0 | -0.196061 | -4.131947 | -1.468040 |

**16A-2**

| | | | | |
|---|---|---|---|---|
| 63 | 0 | -1.037902 | 1.967358 | -0.536785 |
| 32 | 0 | 2.646509 | -2.101316 | -1.358413 |
| 32 | 0 | -2.783808 | 0.029329 | 1.593044 |
| 32 | 0 | -5.296457 | -0.337746 | 0.778704 |
| 32 | 0 | -3.623580 | -2.250832 | 0.386656 |
| 32 | 0 | -1.125246 | -1.121642 | -2.262155 |
| 32 | 0 | -4.105034 | 1.866802 | 0.212636 |
| 32 | 0 | 1.436501 | -1.654905 | 0.880209 |
| 32 | 0 | 2.786085 | -0.406266 | 2.654673 |
| 32 | 0 | -3.415445 | -0.119207 | -1.185309 |
| 32 | 0 | -1.091303 | -1.528802 | 0.168171 |
| 32 | 0 | 4.311076 | -1.198402 | 0.539455 |
| 32 | 0 | 3.864242 | 1.410022 | 0.991097 |
| 32 | 0 | 3.905855 | 0.310581 | -1.575648 |
| 32 | 0 | 1.157223 | 1.053849 | 1.378000 |
| 32 | 0 | 2.195138 | 2.237198 | -0.791025 |
| 32 | 0 | 1.181615 | -0.061900 | -1.353299 |

**16A-3**

| | | | | |
|---|---|---|---|---|
| 63 | 0 | 0.879199 | 2.424834 | 0.222260 |
| 32 | 0 | 3.560411 | 0.528040 | 0.140609 |
| 32 | 0 | -1.312646 | -2.050933 | -0.391133 |
| 32 | 0 | 2.181933 | -1.971524 | 0.411815 |
| 32 | 0 | -2.957099 | -0.378915 | -2.006359 |
| 32 | 0 | -3.943364 | 0.699573 | 0.382952 |
| 32 | 0 | 1.021505 | -2.720274 | -1.630915 |
| 32 | 0 | 1.747015 | -0.382014 | -2.129704 |
| 32 | 0 | -2.580479 | -0.773101 | 2.282116 |
| 32 | 0 | 4.196688 | -1.393893 | 1.615167 |
| 32 | 0 | 3.162494 | 1.644885 | -2.010350 |
| 32 | 0 | -2.360788 | 2.146696 | -1.042965 |
| 32 | 0 | -0.268779 | -0.369294 | 1.240964 |
| 32 | 0 | -0.597836 | 0.281132 | -1.278029 |
| 32 | 0 | 2.085969 | 0.180081 | 2.238067 |
| 32 | 0 | -3.798800 | -1.879400 | 0.091805 |
| 32 | 0 | -1.867148 | 1.665051 | 1.648385 |

**17A-1**

| | | | | |
|---|---|---|---|---|
| 63 | 0 | -0.565758 | 2.571272 | -0.246217 |
| 32 | 0 | 2.510322 | 2.208439 | 0.747014 |
| 32 | 0 | -4.779450 | -0.396961 | 0.081762 |

| | | | | |
|---|---|---|---|---|
| 32 | 0 | 4.217321 | −1.658858 | 0.174426 |
| 32 | 0 | 1.445238 | −1.750726 | −0.771660 |
| 32 | 0 | −3.434705 | −0.906089 | 2.317990 |
| 32 | 0 | −3.333033 | 1.589397 | 1.270333 |
| 32 | 0 | −0.956783 | −0.661441 | −1.597714 |
| 32 | 0 | 1.729976 | 0.903659 | −1.469349 |
| 32 | 0 | 4.374912 | 0.911463 | −0.515539 |
| 32 | 0 | 3.611696 | 0.141810 | 2.042673 |
| 32 | 0 | −3.080082 | 1.199784 | −1.351107 |
| 32 | 0 | 3.335567 | −0.876687 | −2.255491 |
| 32 | 0 | −3.369027 | −1.311224 | −2.129184 |
| 32 | 0 | −1.487938 | −0.121230 | 0.866760 |
| 32 | 0 | −2.805117 | −2.376927 | 0.211747 |
| 32 | 0 | 1.055801 | 0.136155 | 1.143976 |
| 32 | 0 | 2.079136 | −2.092758 | 1.718103 |

**17A-2**

| | | | | |
|---|---|---|---|---|
| 63 | 0 | 1.576506 | 4.780599 | 0.000000 |
| 32 | 0 | −1.096489 | −3.816049 | 1.372550 |
| 32 | 0 | 1.028547 | −4.508136 | 0.000000 |
| 32 | 0 | 0.057685 | 2.625740 | 1.467646 |
| 32 | 0 | −1.705349 | 1.074174 | 2.316266 |
| 32 | 0 | −2.284226 | 1.945903 | 0.000000 |
| 32 | 0 | −1.402785 | 4.308120 | 0.000000 |
| 32 | 0 | 1.508273 | −0.612991 | 0.000000 |
| 32 | 0 | 1.198932 | −2.699197 | 2.110913 |
| 32 | 0 | −0.633726 | −1.032661 | 1.390055 |
| 32 | 0 | −2.590189 | −2.056929 | 0.000000 |
| 32 | 0 | 2.932129 | −2.675881 | 0.000000 |
| 32 | 0 | 2.062400 | 1.884097 | 0.000000 |
| 32 | 0 | −1.096489 | −3.816049 | −1.372550 |
| 32 | 0 | 0.057685 | 2.625740 | −1.467646 |
| 32 | 0 | −1.705349 | 1.074174 | −2.316266 |
| 32 | 0 | 1.198932 | −2.699197 | −2.110913 |
| 32 | 0 | −0.633726 | −1.032661 | −1.390055 |

**17A-3**

| | | | | |
|---|---|---|---|---|
| 63 | 0 | 0.000000 | 0.000000 | 0.000551 |
| 32 | 0 | 0.000000 | 1.832800 | −2.401264 |
| 32 | 0 | −1.481551 | 1.904883 | 1.891618 |
| 32 | 0 | 0.000000 | 3.408330 | 0.047983 |
| 32 | 0 | −2.209454 | 2.166403 | −0.660643 |
| 32 | 0 | −2.144548 | 0.000000 | −2.242594 |
| 32 | 0 | −3.070283 | 0.000000 | 0.579818 |

| | | | | |
|---|---|---|---|---|
| 32 | 0 | 0.000000 | 0.000000 | 3.107131 |
| 32 | 0 | 0.000000 | −1.832800 | −2.401264 |
| 32 | 0 | −1.481551 | −1.904883 | 1.891618 |
| 32 | 0 | 0.000000 | −3.408330 | 0.047983 |
| 32 | 0 | −2.209454 | −2.166403 | −0.660643 |
| 32 | 0 | 1.481551 | −1.904883 | 1.891618 |
| 32 | 0 | 2.209454 | −2.166403 | −0.660643 |
| 32 | 0 | 2.144548 | 0.000000 | −2.242594 |
| 32 | 0 | 3.070283 | 0.000000 | 0.579818 |
| 32 | 0 | 1.481551 | 1.904883 | 1.891618 |
| 32 | 0 | 2.209454 | 2.166403 | −0.660643 |

**18A-1**

| | | | | |
|---|---|---|---|---|
| 63 | 0 | 2.293998 | −0.040427 | 0.000000 |
| 32 | 0 | −1.174214 | 1.328378 | 0.000000 |
| 32 | 0 | 0.867925 | 2.097459 | −1.577822 |
| 32 | 0 | 2.222054 | 3.704326 | 0.000000 |
| 32 | 0 | −1.531817 | 2.691674 | −2.166365 |
| 32 | 0 | −2.037573 | 4.158097 | 0.000000 |
| 32 | 0 | 0.171667 | 4.660922 | −1.407067 |
| 32 | 0 | 0.867925 | 2.097459 | 1.577822 |
| 32 | 0 | −1.531817 | 2.691674 | 2.166365 |
| 32 | 0 | 0.171667 | 4.660922 | 1.407067 |
| 32 | 0 | −0.776329 | −1.175070 | 0.000000 |
| 32 | 0 | 1.118050 | −2.483257 | −1.518443 |
| 32 | 0 | 1.867992 | −4.452393 | 0.000000 |
| 32 | 0 | −1.348991 | −2.376987 | −2.248500 |
| 32 | 0 | −2.385042 | −3.289368 | 0.000000 |
| 32 | 0 | −0.393432 | −4.687001 | −1.391757 |
| 32 | 0 | 1.118050 | −2.483257 | 1.518443 |
| 32 | 0 | −1.348991 | −2.376987 | 2.248500 |
| 32 | 0 | −0.393432 | −4.687001 | 1.391757 |

**18A-2**

| | | | | |
|---|---|---|---|---|
| 63 | 0 | 0.000000 | 0.000000 | 2.407945 |
| 32 | 0 | 1.309918 | 1.243497 | −0.290527 |
| 32 | 0 | 1.399976 | 2.855949 | 1.736908 |
| 32 | 0 | 2.140333 | 3.589764 | −0.785721 |
| 32 | 0 | 0.000000 | 1.993299 | −2.379824 |
| 32 | 0 | 0.000000 | 4.752895 | 0.660260 |
| 32 | 0 | 0.000000 | 4.571302 | −1.972078 |
| 32 | 0 | −1.309918 | 1.243497 | −0.290527 |
| 32 | 0 | −1.399976 | 2.855949 | 1.736908 |
| 32 | 0 | −2.140333 | 3.589764 | −0.785721 |

| | | | | |
|---|---|---|---|---|
| 32 | 0 | −1.309918 | −1.243497 | −0.290527 |
| 32 | 0 | −1.399976 | −2.855949 | 1.736908 |
| 32 | 0 | −2.140333 | −3.589764 | −0.785721 |
| 32 | 0 | 0.000000 | −1.993299 | −2.379824 |
| 32 | 0 | 0.000000 | −4.752895 | 0.660260 |
| 32 | 0 | 0.000000 | −4.571302 | −1.972078 |
| 32 | 0 | 1.309918 | −1.243497 | −0.290527 |
| 32 | 0 | 1.399976 | −2.855949 | 1.736908 |
| 32 | 0 | 2.140333 | −3.589764 | −0.785721 |

**18A-3**

| | | | | |
|---|---|---|---|---|
| 63 | 0 | 0.261065 | −0.090805 | 0.000000 |
| 32 | 0 | −0.478378 | −0.868120 | 3.132051 |
| 32 | 0 | −1.152054 | 1.638258 | 2.484534 |
| 32 | 0 | 1.567836 | 0.821212 | 2.750479 |
| 32 | 0 | −2.706602 | 1.025920 | 0.000000 |
| 32 | 0 | −2.476670 | −1.075406 | 1.550602 |
| 32 | 0 | −1.286008 | 3.200426 | 0.000000 |
| 32 | 0 | 0.883620 | 2.984109 | 1.411283 |
| 32 | 0 | 2.744448 | 1.851150 | 0.000000 |
| 32 | 0 | 1.764210 | −1.861301 | 2.359404 |
| 32 | 0 | 1.637276 | −3.160484 | 0.000000 |
| 32 | 0 | −0.560106 | −3.007871 | 1.371434 |
| 32 | 0 | −0.478378 | −0.868120 | −3.132051 |
| 32 | 0 | −1.152054 | 1.638258 | −2.484534 |
| 32 | 0 | 1.567836 | 0.821212 | −2.750479 |
| 32 | 0 | −2.476670 | −1.075406 | −1.550602 |
| 32 | 0 | 0.883620 | 2.984109 | −1.411283 |
| 32 | 0 | 1.764210 | −1.861301 | −2.359404 |
| 32 | 0 | −0.560106 | −3.007871 | −1.371434 |

**19A-1**

| | | | | |
|---|---|---|---|---|
| 32 | 0 | −0.250892 | 4.852739 | 1.467963 |
| 32 | 0 | 1.937359 | 5.024181 | 0.000000 |
| 63 | 0 | −2.421233 | 0.461883 | 0.000000 |
| 32 | 0 | 0.715454 | −1.074769 | 0.000000 |
| 32 | 0 | −2.385733 | −3.947950 | 0.000000 |
| 32 | 0 | 1.821620 | −4.657167 | 0.000000 |
| 32 | 0 | −0.718779 | 2.374822 | 2.111799 |
| 32 | 0 | 1.788628 | 2.960424 | 1.603701 |
| 32 | 0 | 0.467133 | 1.430080 | 0.000000 |
| 32 | 0 | −1.155723 | −2.118176 | 1.460748 |
| 32 | 0 | 1.286859 | −2.791557 | 2.082004 |
| 32 | 0 | −0.331806 | −4.723154 | 1.454430 |

| | | | | |
|---|---|---|---|---|
| 32 | 0 | 2.959205 | −2.338030 | 0.000000 |
| 32 | 0 | −1.984808 | 3.544126 | 0.000000 |
| 32 | 0 | −0.250892 | 4.852739 | −1.467963 |
| 32 | 0 | −0.718779 | 2.374822 | −2.111799 |
| 32 | 0 | 1.788628 | 2.960424 | −1.603701 |
| 32 | 0 | −1.155723 | −2.118176 | −1.460748 |
| 32 | 0 | 1.286859 | −2.791557 | −2.082004 |
| 32 | 0 | −0.331806 | −4.723154 | −1.454430 |

**19A-2**

| | | | | |
|---|---|---|---|---|
| 63 | 0 | 2.855281 | −0.455760 | 0.000000 |
| 32 | 0 | −1.648581 | −1.582135 | 0.000000 |
| 32 | 0 | 0.623208 | −1.972353 | 1.438997 |
| 32 | 0 | 2.125666 | −3.602698 | 0.000000 |
| 32 | 0 | −1.592749 | −2.929512 | 2.222061 |
| 32 | 0 | −2.080614 | −4.319463 | 0.000000 |
| 32 | 0 | 0.206665 | −4.721333 | 1.382525 |
| 32 | 0 | −1.856707 | 1.139117 | 0.000000 |
| 32 | 0 | 0.449172 | 1.192516 | 1.380877 |
| 32 | 0 | 2.227985 | 2.655293 | 0.000000 |
| 32 | 0 | −1.573364 | 2.667516 | 2.150461 |
| 32 | 0 | −1.765282 | 4.143324 | 0.000000 |
| 32 | 0 | 0.591563 | 4.001892 | 1.484037 |
| 32 | 0 | −0.032792 | 5.986389 | 0.000000 |
| 32 | 0 | 0.623208 | −1.972353 | −1.438997 |
| 32 | 0 | −1.592749 | −2.929512 | −2.222061 |
| 32 | 0 | 0.206665 | −4.721333 | −1.382525 |
| 32 | 0 | 0.449172 | 1.192516 | −1.380877 |
| 32 | 0 | −1.573364 | 2.667516 | −2.150461 |
| 32 | 0 | 0.591563 | 4.001892 | −1.484037 |

**19A-3**

| | | | | |
|---|---|---|---|---|
| 32 | 0 | 2.408937 | 1.654323 | −1.525815 |
| 63 | 0 | 0.022421 | −0.002658 | −0.000000 |
| 32 | 0 | 1.763894 | −3.042607 | −0.000000 |
| 32 | 0 | −2.178622 | −1.696146 | 1.674672 |
| 32 | 0 | 0.431002 | 0.040708 | −3.108424 |
| 32 | 0 | −2.178622 | −1.696146 | −1.674672 |
| 32 | 0 | 2.555457 | −1.016558 | −1.599291 |
| 32 | 0 | −0.903547 | −3.350963 | 0.000000 |
| 32 | 0 | 0.328508 | −2.545374 | −2.235198 |
| 32 | 0 | −1.662516 | 2.821734 | 0.000000 |
| 32 | 0 | −0.037839 | 2.589548 | −2.267687 |
| 32 | 0 | −3.172530 | 0.281740 | 0.000000 |

| | | | | |
|---|---|---|---|---|
| 32 | 0 | -2.062812 | 0.944984 | -2.299961 |
| 32 | 0 | 2.408937 | 1.654323 | 1.525815 |
| 32 | 0 | 0.431002 | 0.040708 | 3.108424 |
| 32 | 0 | 0.328508 | -2.545374 | 2.235198 |
| 32 | 0 | 2.555457 | -1.016558 | 1.599291 |
| 32 | 0 | 1.041297 | 3.352359 | -0.000000 |
| 32 | 0 | -2.062812 | 0.944984 | 2.299961 |
| 32 | 0 | -0.037839 | 2.589548 | 2.267687 |

**20A-1**

| | | | | |
|---|---|---|---|---|
| 63 | 0 | 2.472973 | -0.997733 | 0.000000 |
| 32 | 0 | -1.464421 | 1.551170 | 2.315074 |
| 32 | 0 | 1.337358 | 0.873594 | 2.311425 |
| 32 | 0 | 1.427833 | -1.739932 | 2.763077 |
| 32 | 0 | 0.228925 | 2.134264 | 4.207923 |
| 32 | 0 | -1.611072 | 0.265950 | 4.824649 |
| 32 | 0 | 0.982389 | -0.383474 | 4.911281 |
| 32 | 0 | -0.621121 | -0.964419 | 1.352685 |
| 32 | 0 | -2.773986 | -0.672850 | 2.638475 |
| 32 | 0 | -0.851468 | -2.115337 | 4.011426 |
| 32 | 0 | 2.126706 | 2.208970 | 0.000000 |
| 32 | 0 | -0.304245 | 1.857385 | -0.000000 |
| 32 | 0 | -1.464421 | 1.551170 | -2.315074 |
| 32 | 0 | 1.337358 | 0.873594 | -2.311425 |
| 32 | 0 | 1.427833 | -1.739932 | -2.763077 |
| 32 | 0 | 0.228925 | 2.134264 | -4.207923 |
| 32 | 0 | -1.611072 | 0.265950 | -4.824649 |
| 32 | 0 | 0.982389 | -0.383474 | -4.911281 |
| 32 | 0 | -0.621121 | -0.964419 | -1.352685 |
| 32 | 0 | -2.773986 | -0.672850 | -2.638475 |
| 32 | 0 | -0.851468 | -2.115337 | -4.011426 |

**20A-2**

| | | | | |
|---|---|---|---|---|
| 63 | 0 | 2.424603 | -0.092133 | 0.000000 |
| 32 | 0 | -0.571472 | -1.365330 | 0.000000 |
| 32 | 0 | 1.190074 | -2.580353 | 1.475126 |
| 32 | 0 | 2.342377 | -4.433029 | 0.000000 |
| 32 | 0 | -1.268102 | -3.108313 | 2.070693 |
| 32 | 0 | -1.915759 | -4.915818 | 0.000000 |
| 32 | 0 | 0.239009 | -5.143107 | 1.437714 |
| 32 | 0 | -0.715153 | 1.327572 | 0.000000 |
| 32 | 0 | 1.301731 | 2.439255 | 1.384679 |
| 32 | 0 | 1.846798 | 4.601053 | 0.000000 |
| 32 | 0 | -1.560470 | 2.485295 | 2.042538 |

| | | | | |
|---|---|---|---|---|
| 32 | 0 | −2.105031 | 4.031874 | 0.000000 |
| 32 | 0 | −0.023506 | 4.567686 | 2.003032 |
| 32 | 0 | −0.535865 | 6.108631 | 0.000000 |
| 32 | 0 | −2.876804 | −2.494493 | 0.000000 |
| 32 | 0 | 1.190074 | −2.580353 | −1.475126 |
| 32 | 0 | −1.268102 | −3.108313 | −2.070693 |
| 32 | 0 | 0.239009 | −5.143107 | −1.437714 |
| 32 | 0 | 1.301731 | 2.439255 | −1.384679 |
| 32 | 0 | −1.560470 | 2.485295 | −2.042538 |
| 32 | 0 | −0.023506 | 4.567686 | −2.003032 |

**20A-3**

| | | | | |
|---|---|---|---|---|
| 32 | 0 | 0.000000 | 3.252783 | 0.000000 |
| 32 | 0 | 0.000000 | 1.894996 | 2.747258 |
| 63 | 0 | 0.000000 | 0.000000 | 0.000000 |
| 32 | 0 | 2.199683 | 2.243209 | 1.345322 |
| 32 | 0 | 3.243597 | −0.000000 | −0.000000 |
| 32 | 0 | 2.002910 | −0.000000 | 2.692144 |
| 32 | 0 | −0.000000 | −3.252783 | 0.000000 |
| 32 | 0 | 0.000000 | 1.894996 | −2.747258 |
| 32 | 0 | −0.000000 | −1.894996 | −2.747258 |
| 32 | 0 | −0.000000 | −1.894996 | 2.747258 |
| 32 | 0 | 2.199683 | 2.243209 | −1.345322 |
| 32 | 0 | 2.199683 | −2.243209 | −1.345322 |
| 32 | 0 | 2.199683 | −2.243209 | 1.345322 |
| 32 | 0 | 2.002910 | 0.000000 | −2.692144 |
| 32 | 0 | −2.199683 | 2.243209 | 1.345322 |
| 32 | 0 | −3.243597 | 0.000000 | −0.000000 |
| 32 | 0 | −2.002910 | 0.000000 | 2.692144 |
| 32 | 0 | −2.199683 | 2.243209 | −1.345322 |
| 32 | 0 | −2.199683 | −2.243209 | −1.345322 |
| 32 | 0 | −2.199683 | −2.243209 | 1.345322 |
| 32 | 0 | −2.002910 | −0.000000 | −2.692144 |

Table S2. Total energies at mPW2PLYP with zero-point energy at TPSSh (in Hartree).

| Cluster | Energy | ZPVE | Total | Cluster | Energy | ZPVE | Total |
|---|---|---|---|---|---|---|---|
| 7A-1 | -15246.95000 | 0.00625 | -15246.94375 | 14A-1 | -29784.13459 | 0.01287 | -29784.12171 |
| 7A-2 | -15246.94060 | 0.00590 | -15246.93470 | 14A-2 | -29784.13294 | 0.01289 | -29784.12006 |
| 7A-3 | -15246.93945 | 0.00589 | -15246.93356 | 14A-3 | -29784.12863 | 0.01278 | -29784.11585 |
| 8A-1 | -17323.69886 | 0.00764 | -17323.69122 | 15A-1 | -31860.88016 | 0.01341 | -31860.86675 |
| 8A-2 | -17323.68707 | 0.00685 | -17323.68023 | 15A-2 | -31860.87199 | 0.01357 | -31860.85841 |
| 8A-3 | -17323.68078 | 0.00678 | -17323.67399 | 15A-3 | -31860.86237 | 0.01369 | -31860.84868 |
| 9A-1 | -19400.46204 | 0.00815 | -19400.45389 | 16A-1 | -33937.6091 | 0.01481 | -33937.59429 |
| 9A-2 | -19400.44841 | 0.00807 | -19400.44035 | 16A-2 | -33937.59656 | 0.01444 | -33937.58212 |
| 9A-3 | -19400.43396 | 0.00811 | -19400.42586 | 16A-3 | -33937.59207 | 0.01474 | -33937.57733 |
| 10A-1 | -21477.19562 | 0.00928 | -21477.18634 | 17A-1 | -36014.33526 | 0.01519 | -36014.32008 |
| 10A-2 | -21477.19317 | 0.00925 | -21477.18392 | 17A-2 | -36014.32563 | 0.01607 | -36014.30957 |
| 10A-3 | -21477.17083 | 0.00903 | -21477.16180 | 17A-3 | -36014.29778 | 0.01421 | -36014.28357 |
| 11A-1 | -23553.90906 | 0.01001 | -23553.89905 | 18A-1 | -38091.09147 | 0.01665 | -38091.07482 |
| 11A-2 | -23553.90157 | 0.00968 | -23553.89189 | 18A-2 | -38091.08558 | 0.01645 | -38091.06913 |
| 11A-3 | -23553.89927 | 0.00946 | -23553.88980 | 18A-3 | -38091.03503 | 0.01506 | -38091.01996 |
| 11A-4 | -23553.89459 | 0.00974 | -23553.88485 | 19A-1 | -40167.83417 | 0.01760 | -40167.81657 |
| 12A-1 | -25630.64312 | 0.01079 | -25630.63233 | 19A-2 | -40167.83258 | 0.01760 | -40167.81498 |
| 12A-2 | -25630.63633 | 0.01052 | -25630.62582 | 19A-3 | -40167.77298 | 0.01551 | -40167.75747 |
| 13A-1 | -27707.39775 | 0.01199 | -27707.38576 | 20A-1 | -42244.56782 | 0.01847 | -42244.54935 |
| 13A-2 | -27707.38998 | 0.01212 | -27707.37786 | 20A-2 | -42244.55904 | 0.01840 | -42244.54064 |
| 13A-3 | -27707.38637 | 0.01183 | -27707.37453 | 20A-3 | -42244.52883 | 0.01644 | -42244.51239 |

Table S3 The dipole moment ($\mu_0$, in a.u.), static polarizability ($\alpha_0$, in a.u.), static first hyperpolarizability ($\beta_{tot}$, in a.u.), the components of hyperpolarizability in the x, y, and z directions ($\beta_{xxx}$ $\beta_{yyy}$ $\beta_{zzz}$, in a.u.) as well as the projection of the hyperpolarizability in the direction of the dipole moment ($\beta_{prj}$, in a.u.) of $EuGe_n^-$ ($n$ = 7–20) with BS4(aug-cc-pVTZ for Ge and ma-def2-TZVP for Eu)

| *n.* | $\mu_0$ | $\alpha_0$ | $\beta_{prj}$ | $\beta_{tot}$ | $\beta_{xxx}$ | $\beta_{yyy}$ | $\beta_{zzz}$ |
|---|---|---|---|---|---|---|---|
| 7 | 2.80 | 667.31 | -24160.89 | 27414.39 | 26202.80 | 8059.90 | -0.00 |
| 8 | 0.83 | 514.87 | -35220.36 | 35220.36 | -0.00 | -0.00 | -35220.36 |
| 9 | 1.97 | 565.64 | -14839.48 | 14839.48 | 151.87400 | -14838.70 | 0.00 |
| 10 | 1.35 | 575.88 | -35839.30 | 35839.35 | -22742.67 | -27698.92 | 0.00 |
| 11 | 3.94 | 528.67 | -22437.51 | 22494.81 | 19098.51 | 11818.54 | -1259.19 |
| 12 | 3.47 | 552.89 | -12781.24 | 12794.49 | -6011.51 | -11293.99 | 80.29 |
| 13 | 3.31 | 819.32 | -797375.13 | 819740.27 | 413142.50 | -689678.50 | 160096.70 |
| 14 | 2.27 | 630.31 | -2750.65 | 3135.48 | -1225.75 | -2885.96 | 0.00 |
| 15 | 3.49 | 680.24 | -100681.12 | 100681.12 | 0.00 | 0.00 | -100681.12 |
| 16 | 2.98 | 682.23 | -2373.54 | 2565.37 | 2552.83 | -253.35 | 0.00 |
| 17 | 2.84 | 597.05 | -2691.23 | 2784.02 | -131.20 | -2778.45 | 117.44 |
| 18 | 2.47 | 750.14 | -2520.85 | 2555.01 | -1866.98 | 1744.26 | 0.00 |
| 19 | 2.71 | 788.62 | -2681.47 | 2733.60 | 2622.77 | 770.47 | 0.00 |
| 20 | 2.52 | 860.61 | -3453.97 | 3489.03 | -3161.18 | 1476.57 | 0.00 |

Table S4. The main transitions, oscillator strengths($f_0$, in a.u.), difference in dipole moment($\Delta\mu$, in a.u.)transition energies($\Delta E$, in eV), wavelengths(λ, in nm) and orbital contribution of the crucial excited states for the $EuGe_n^-$ ($n$ = 7–20) cluster.

| Cluster | Transitions | $f_0$ | $\Delta\mu$ | $\Delta E$ | Wavelengths | The orbital contribution of the crucial transitions |
|---|---|---|---|---|---|---|
| 7 | $N_0 \rightarrow N_5$ | 0.1114 | 2.8954 | 1.57 | 789.71 | H(α)→ L+2(α) 82.8% |
| 8 | $N_0 \rightarrow N_5$ | 0.1858 | 4.5664 | 1.66 | 746.62 | H(α) → L+4(α) 86.0%, H(α) → L+6(α) 6.3% |
| | $N_0 \rightarrow N_{21}$ | 0.1273 | 2.3087 | 2.25 | 550.87 | H(α) → L+6(α) 59.6%, H(α) → L+13(α) 7.1% |
| 9 | $N_0 \rightarrow N_2$ | 0.2878 | 8.4229 | 1.39 | 888.90 | H(α) → L(α) 96.2% |
| 10 | $N_0 \rightarrow N_{30}$ | 0.0963 | 1.4872 | 2.64 | 468.94 | H(α) → L+9(α) 43.1%, H-2(α) → L+2(α) 18.4% |
| | $N_0 \rightarrow N_{29}$ | 0.0941 | 1.4649 | 2.62 | 472.88 | H(α) → L+9(α) 33.5%, H-2(α) → L+2(α) 22.7% |
| 11 | $N_0 \rightarrow N_6$ | 0.0359 | 1.1425 | 1.28 | 966.66 | H(β) → L+1(β) 66.7%, H-1(α) → L(α) 26.1% |
| 12 | $N_0 \rightarrow N_{24}$ | 0.0232 | 0.4485 | 2.11 | 586.35 | H-2(β) → L+1(β) 53.5%, H-3(α) → L(α) 12.0% |
| 13 | $N_0 \rightarrow N_1$ | 0.0504 | 4.5014 | 0.46 | 2710.63 | H(α) → L(α) 97.3% |
| | $N_0 \rightarrow N_{37}$ | 0.0214 | 2.5083 | 2.51 | 494.30 | H(α) → L+12(α) 35.0%,H-3(α) → L+1(α) 14.3% |
| 14 | $N_0 \rightarrow N_{25}$ | 0.0157 | 0.2685 | 2.39 | 519.46 | H-1(β) → L+1(β) 23.7%, H-2 (α) → L(α) 20.3%, H-12(β) → L(β) 14.3% |
| | $N_0 \rightarrow N_{32}$ | 0.0146 | 0.2322 | 2.56 | 483.78 | H-1(β) → L+1(β) 21.5%, H-12(β) → L(β) 20.0% |
| 15 | $N_0 \rightarrow N_{51}$ | 0.0382 | 0.6204 | 2.51 | 493.18 | H(α) → L+15(α) 59.6%, H(α) → L+14(α) 28.5% |
| | $N_0 \rightarrow N_2$ | 0.0194 | 1.5755 | 0.50 | 2464.89 | H(α) → L(α) 98.8% |
| 16 | $N_0 \rightarrow N_8$ | 0.0179 | 0.4294 | 1.71 | 726.84 | H(α) → L+1(α) 46.7%, H-5(β) → L(β) 41.1% |
| | $N_0 \rightarrow N_{26}$ | 0.0141 | 0.2477 | 2.32 | 533.91 | H(α) → L+4(α) 43.2%, H-11(β) → L(β) 11.6% |
| 17 | $N_0 \rightarrow N_{23}$ | 0.0112 | 0.2218 | 2.06 | 601.43 | H(α) → L+4(α) 49.3%, H(β) → L+2(β) 16.4% |
| 18 | $N_0 \rightarrow N_{18}$ | 0.0355 | 0.7709 | 1.88 | 660.16 | H(α) → L+1(α) 78.2% |
| | $N_0 \rightarrow N_{29}$ | 0.0310 | 0.5853 | 2.16 | 573.58 | H-5(β) → L+1(β) 17.0%, H-8(β) → L(β) 15.5%, H-3(β) → L+1(β) 15.1%, H-7(α) → L(α) 13.7% |
| 19 | $N_0 \rightarrow N_{38}$ | 0.0480 | 0.8030 | 2.44 | 508.30 | H-2(β) → L+1(β) 26.9%, H-3(α) → L(α) 23.3% |
| 20 | $N_0 \rightarrow N_{56}$ | 0.0414 | 0.6604 | 2.56 | 484.56 | H-4(β) → L+2(β) 26.9%, H-5(α) → L+1(α) 12.6%, H-6(α) → L(α) 10.3%, |
